# Supplementary material for: Probing the molecular structure at graphite–water interfaces by correlating 3D-AFM and SHINERS
Source: Nat Commun. 2026 Jan 31;17:2230. doi: 10.1038/s41467-026-68667-y (PMC12963383; doi:10.1038/s41467-026-68667-y)
Supplement: Supplementary file 1 — Supplementary information [file 41467_2026_68667_MOESM1_ESM.pdf]

Supplementary Information

Probing the molecular structure at graphite–water interfaces by correlating 3D-AFM and SHINERS

Lalith Krishna Samanth Bonagiri[1,2]†, Diana M. Arvelo[3]†, Fujia Zhao[1,4]†, Jaehyeon Kim[1,4], Qian Ai[1,4], Shan Zhou[1,4], Kaustubh S. Panse[1,4], Ricardo Garcia[3]*, and Yingjie Zhang[1,4,5]*

1. Materials Research Laboratory, University of Illinois, Urbana, IL 61801, USA

2. Department of Mechanical Science and Engineering, University of Illinois, Urbana, IL 61801, USA

3. Instituto de Ciencia de Materiales de Madrid, CSIC, Madrid 28049, Spain

4. Department of Materials Science and Engineering, University of Illinois, Urbana, IL 61801, USA

5. Beckman Institute for Advanced Science and Technology, University of Illinois, Urbana, IL 61801, USA

†These authors contributed equally

*Correspondence to: yjz@illinois.edu (Y.Z.) and r.garcia@csic.es (R.G.)

Supplementary Notes

Supplementary Note 1

Using the Hertz contact mechanics model, the deformation (indentation) of the graphite substrate under our imaging conditions can be estimated. The effective elastic modulus E^* for a silicon tip in contact with graphite is[1]

$$\frac{1}{E^*} = \frac{1-\nu_\mathrm{t}^2}{E_\mathrm{t}} + \frac{1-\nu_\mathrm{s}^2}{E_\mathrm{s}} \tag{1}$$

where Young's modulus $E_\mathrm{t} \approx 160$ GPa and Poisson ratio $\nu_\mathrm{t} \approx 0.278$ for silicon[2,3]. For graphite, these values are $E_\mathrm{s} \approx 36$ GPa and $\nu_\mathrm{s} \approx 0.24$[3,4]. Inserting these values into the above formula, we obtain $E^* \approx 31$ GPa. For a spherical tip of radius R under a normal load F, the indentation δ is given by the Hertz relation[1]:

$$\delta = \left(\frac{3F}{4E^*\sqrt{R}}\right)^{\frac{2}{3}} \tag{2}$$

Using representative 3D-AFM parameters (tip radius $R \approx 5$ nm, maximum load of $F \approx 500$ pN), the indentation depth is $\delta \approx 0.31$ Å. This value is substantially smaller than the interlayer distance of pristine interfacial water (≈3 Å).

Supplementary Note 2

According to Gouy-Chapman-Stern (GCS) theory, the electric field near the electrode is screened within a few Å when a large electrode bias is applied ($|\phi_0| > \approx 1$ V). To estimate the electrostatic stabilization of interfacial water under such conditions, we followed the standard formalism in Bard and Faulkner[5]. The electrostatic potential profile $\phi(z)$ near the electrode surface is separated into two regions: the Stern layer $(0 < z < z_2)$ and the diffuse layer $(z > z_2)$.

In the compact Stern layer, the potential is assumed to drop linearly:

$$\phi(z) = \phi_2 - \left(\frac{\mathrm{d}\phi}{\mathrm{d}z}\right)\Big|_{z=z_2} (z_2 - z), \quad z < z_2 \tag{3}$$

where $\phi_2 = \phi(z_2)$. From GCS theory, the electric field strength at z_2 for a symmetric 1:1 electrolyte is given by:

$$\left(\frac{\mathrm{d}\phi}{\mathrm{d}z}\right)\Big|_{z=z_2} = -\sqrt{\frac{8\mathrm{k_B}Tn_0}{\varepsilon\varepsilon_0}} \cdot \sinh\left(\frac{e\phi_2}{2\mathrm{k_B}T}\right) \tag{4}$$

Here $\mathrm{k_B}$ is the Boltzmann constant, T is temperature, e is the elementary charge, ε is the dielectric constant of water, ε_0 is the vacuum permittivity and n_0 is the bulk number concentration of each ion.

Beyond the Stern layer, the potential drop is described by:

$$\phi(z) = \frac{4\mathrm{k_B}T}{e} \cdot \tanh^{-1}\left[\exp\big(-K(z - z_2)\big) \cdot \tanh\left(\frac{e\phi_2}{4\mathrm{k_B}T}\right)\right], \quad z > z_2 \tag{5}$$

The inverse Debye length K for a dilute aqueous electrolyte (ε =78.49) at 25 °C is given by:

$$K = 3.29 \times 10^7 \sqrt{C^*} \times 100\ \mathrm{m}^{-1} \tag{6}$$

where C^* is the bulk electrolyte concentration in mol L^{-1}.

Using this framework, we computed the potential and electric field profiles (see Supplementary Fig. 32) for a 1:1 electrolyte at 0.01 M concentration, assuming z_2 = 3 Å and a ϕ_2 of −0.29 V (such that $\phi_0 \approx -1$ V). The calculated electric field at z_2 is $E \approx -2.4 \times 10^9$ V m^{-1}. Given the dipole moment of water (μ = 9.8 $\times 10^{-30}$ C m)[6], the electrostatic free energy change of the interfacial water is estimated as:

$$\Delta G = -\left|\vec{\mu} \cdot \vec{E}\right| \approx -3.38\ \mathrm{kcal\ mol^{-1}} \tag{7}$$

This energy is comparable to the free energy decrease associated with hydrocarbon accumulation at a neutral graphite–water interface[7]. As a result, under strong negative polarization, water might be electrostatically stabilized at the interface, making hydrocarbon accumulation less favorable at electrified graphite–water interfaces compared to the uncharged interface.

Supplementary Figures

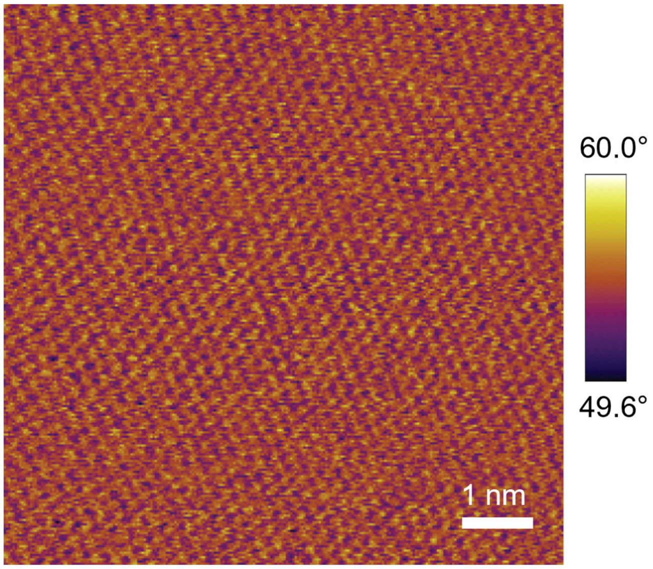

Supplementary Fig. 1 | AFM phase image of a pristine HOPG surface immersed in water. All 3D-AFM measurements in this work were conducted in regions similar to the one depicted here. The lattice-resolution image reveals a hexagonal lattice with a lattice constant of ≈2.5 Å. The image was obtained hundreds of nm away from any step edge. Imaging was performed at Madrid, using PPP-FM cantilevers (Nanosensors) operated at the second eigenmode (AC, amplitude modulation mode). Experimental parameters: free amplitude, 200 pm; setpoint amplitude, 150 pm; resonant frequency, 195.652 kHz; spring constant, 100 N m^{-1}; quality factor, 10.5.

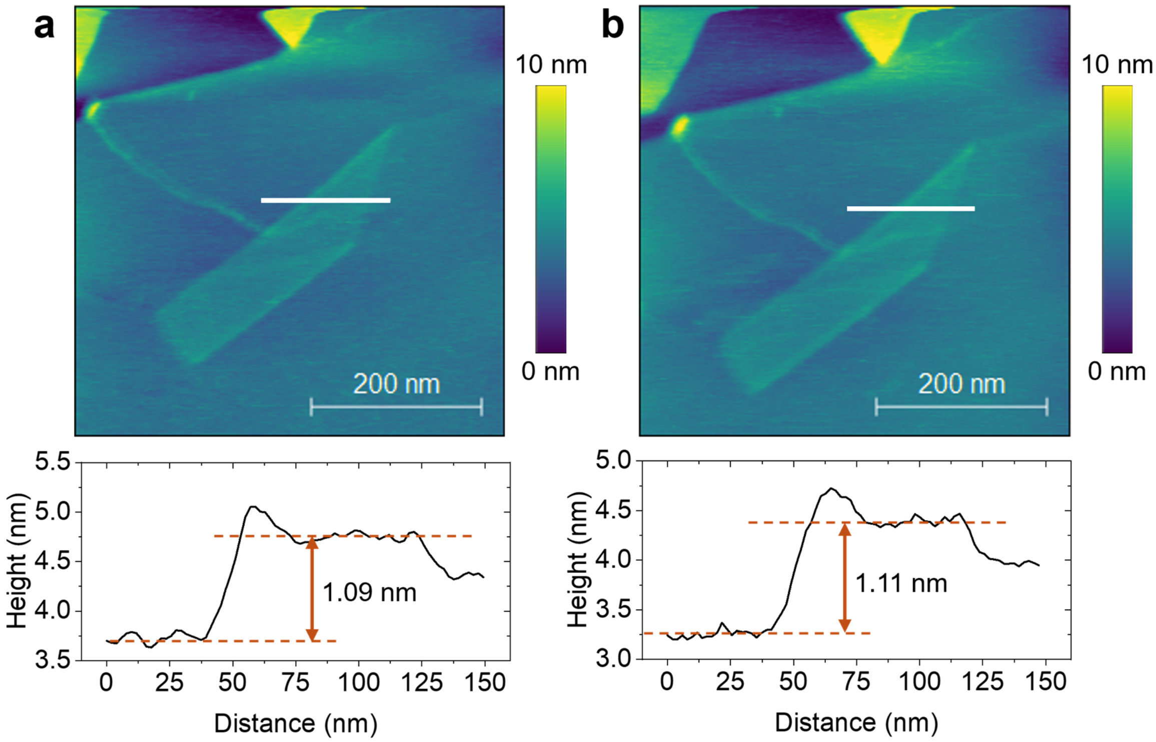

Supplementary Fig. 2 | Large-area AFM images of HOPG surface in aqueous solution. Location: Madrid. Electrolyte: 200 mM K_2SO_4 in water. HOPG surface topography maps were measured at 0 V, (**a**) before and (**b**) after applying −1 V vs. Pt for 2 min. The cross-sectional height profiles are shown below the corresponding images, indicating no change of the step height. Imaging mode: AC, amplitude modulation, first eigenmode. AFM cantilever: ArrowUHFAuD (Nanosensors). Imaging parameters: resonant frequency, 423.94 kHz; spring constant, 5.35 N m^{-1}; quality factor, 4.2; free amplitude, 3.8 nm; setpoint amplitude, 2.73 nm. Source data are provided as a Source Data file.

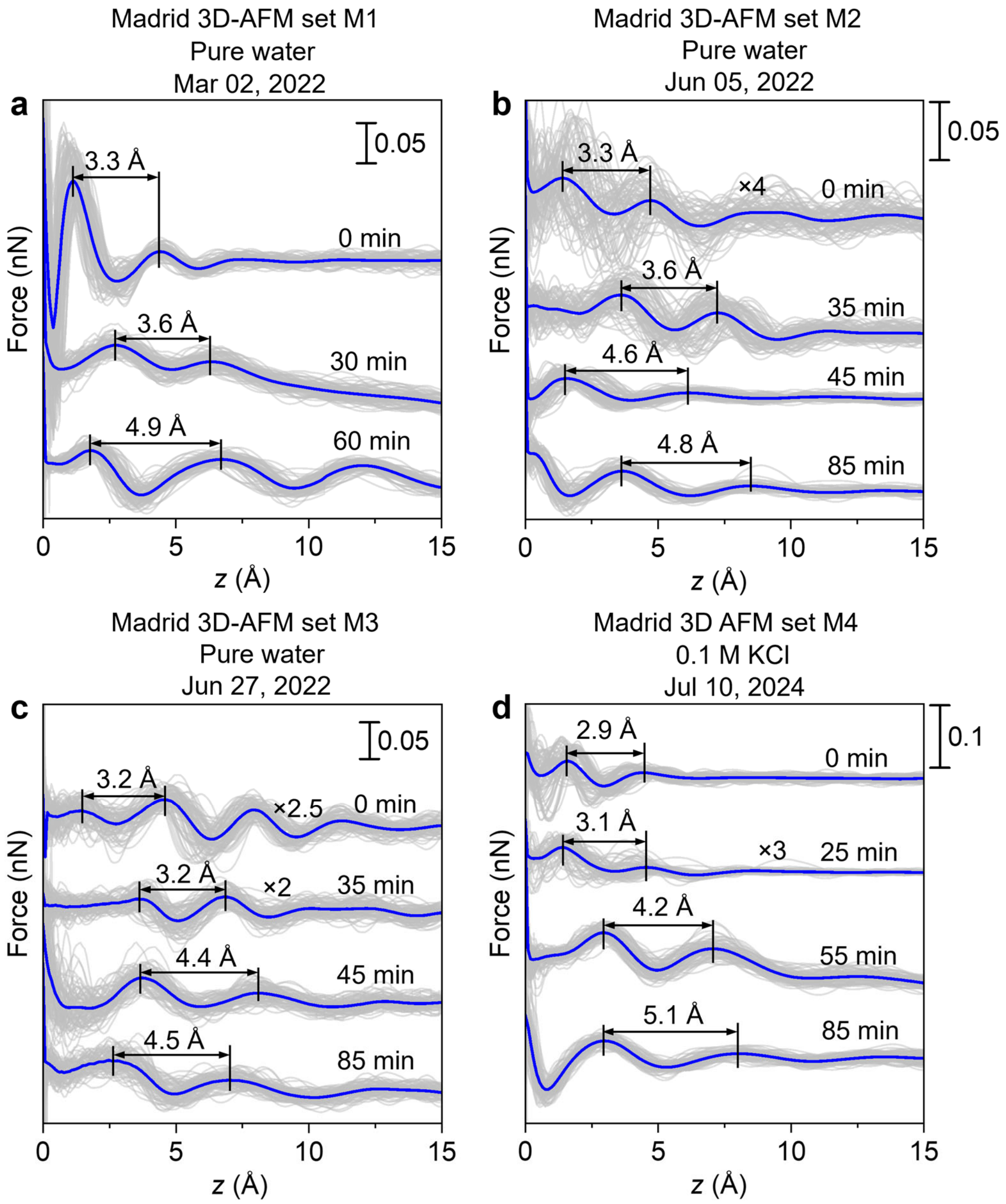

Supplementary Fig. 3 | Time evolution of 3D-AFM force–distance curves at the graphite–water interface at OCP. a–d, Sets of 3D-AFM force–distance curves recorded at different date and time as marked, for HOPG/pure water (**a**–**c**) and HOPG/0.1 M KCl in water (**d**). Gas environment: air, open cell. These measurements were performed at Madrid. Individual curves are shown in gray, while averaged curves are in blue. d_{12}, as marked on the curves, reproducibly increased from ≈3 Å to ≈4–5 Å after ≈1 hour of ageing. Note that the curves in data set M1 at 0 minutes and 60 minutes (**a**) and set M4 at 0 minutes and 85 minutes (**d**) are the same as the corresponding ones in Fig. 2b (pure water and 0.1 M KCl panels) of the main article. Additionally, the curves in set M1 at 0 minutes and 60 minutes (**a**) and set M3 at 0 minutes (**c**) are the same as the corresponding ones in Supplementary Fig. 4a. Source data are provided as a Source Data file.

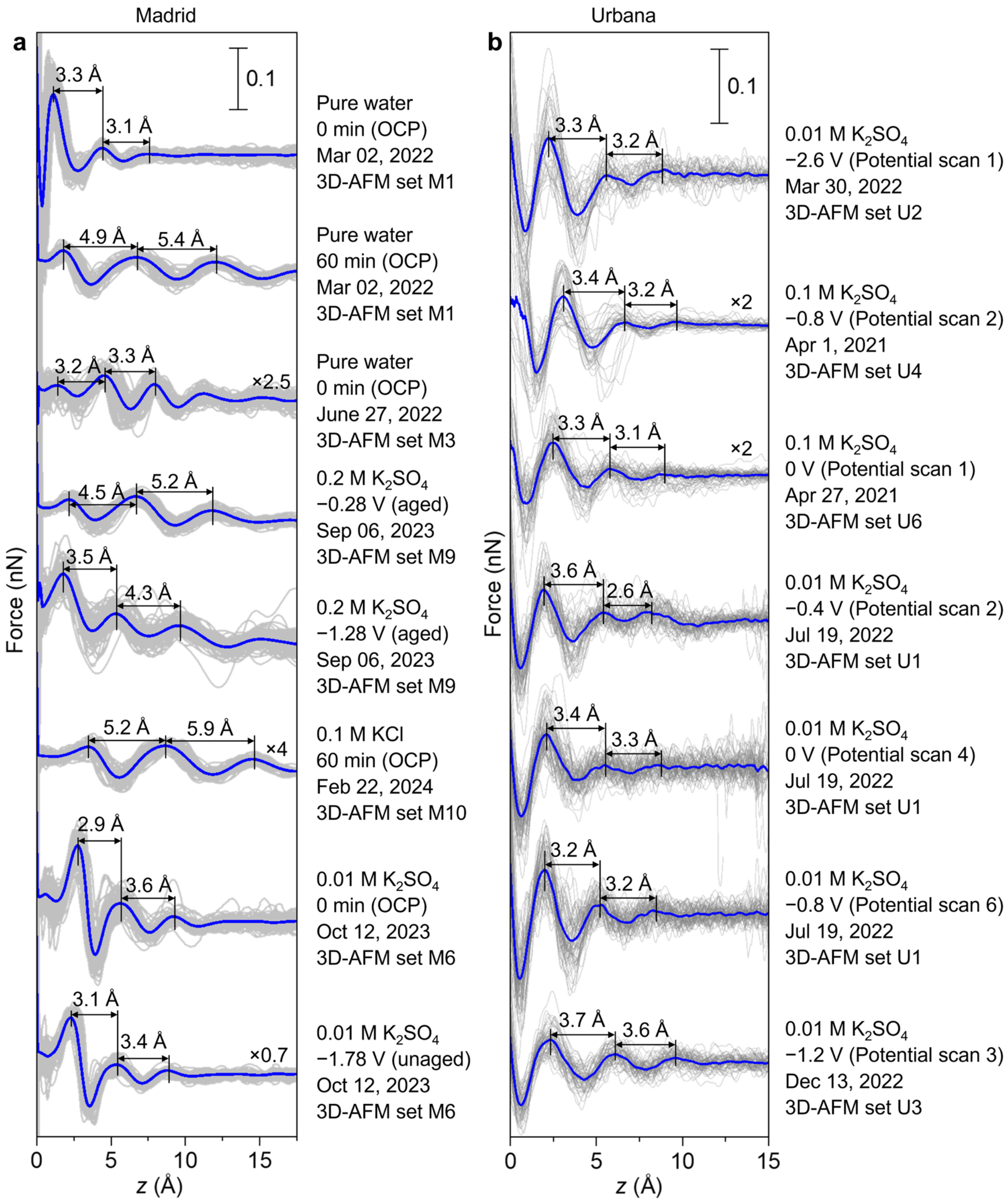

Supplementary Fig. 4 | Gallery of force–distance curves depicting multiple layers for a series of water/electrolytes on HOPG at various conditions. Results in (**a**) were obtained in Madrid, and (**b**) in Urbana. Raw curves: gray; average curve: blue. The 1st–2nd layer distance (d_{12}) and 2nd–3rd layer distance (d_{23}) are marked on the figure. Experimental conditions, including water/electrolyte composition/concentration, electrode potential, ageing condition, and measurement date, are marked alongside each set of force–distance curves. The curves shown in (**a**) at 0 minutes and 60 minutes dated Mar 02, 2022 (Madrid 3D-AFM set M1) are the same as the corresponding ones in Fig. 2b (pure water panel) of the main article. Each force–distance curve shown in this figure also appears in other supplementary figures (identifiable by their labels).

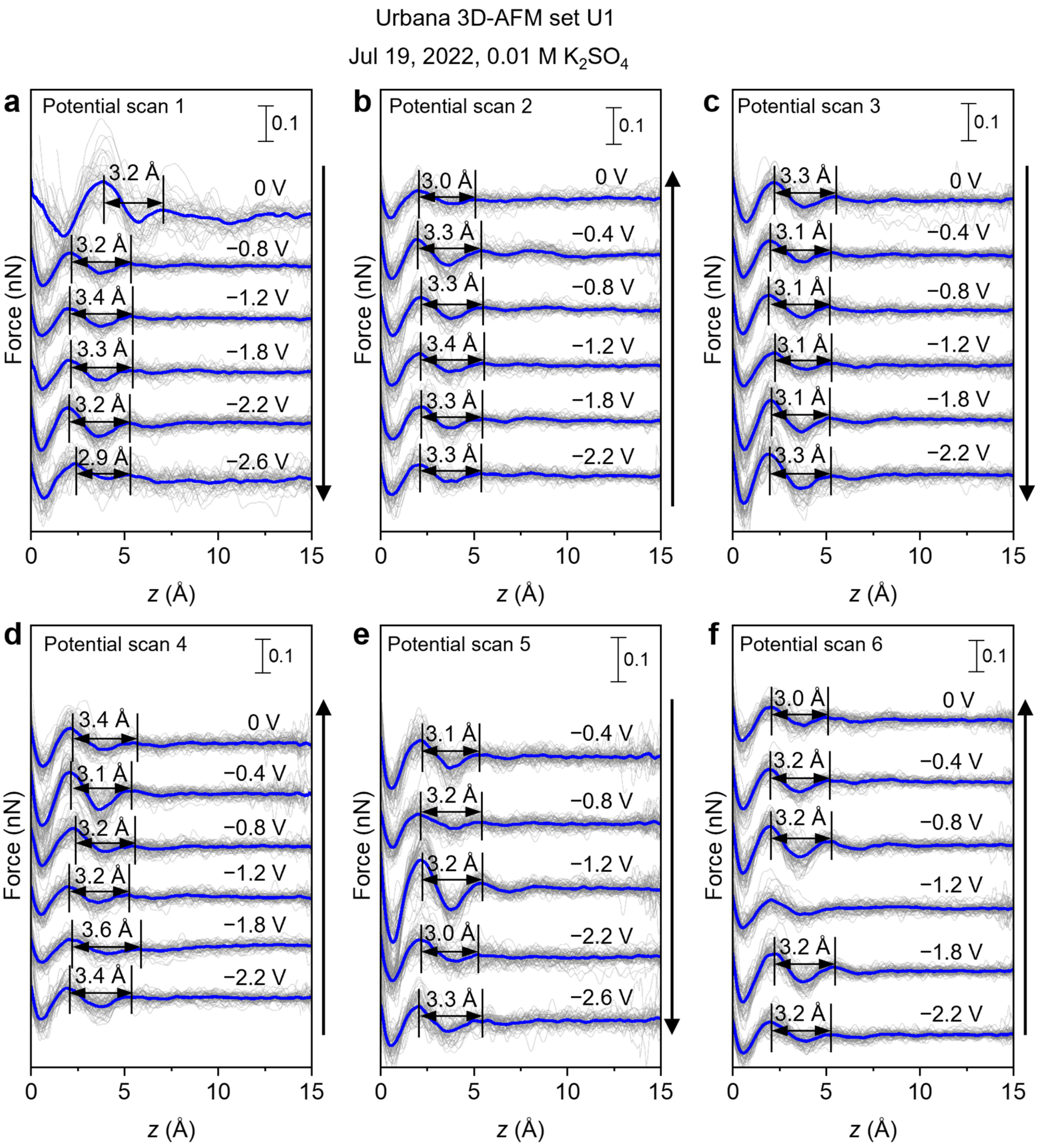

Supplementary Fig. 5 | Effect of electrode potential on 3D-AFM force–distance curves at a pristine HOPG/aqueous electrolyte interface. These measurements were performed on July 19th, 2022, in an argon-sealed cell (Urbana, 3D AFM data set U1). Electrolyte: 0.01 M K_2SO_4 in water. Gray: average curves of single *x*–*z* maps, blue: average of the gray curves. Vertical, panel-spanning arrows indicate the potential scan direction. The surface remained pristine throughout the measurement, as indicated by the nearly constant ≈3–3.5 Å interlayer distance at all the applied potentials throughout multiple potential scans. Note that the curves in panel (**c**) are the same as those in Fig. 3a of the main article. Supplementary Figs. 6–10 show additional potential-dependent 3D-AFM results measured in Urbana at different dates, for HOPG/0.01 M (or 0.1 M) K_2SO_4 in water samples prepared under nominally identical conditions as those in this figure. Source data are provided as a Source Data file.

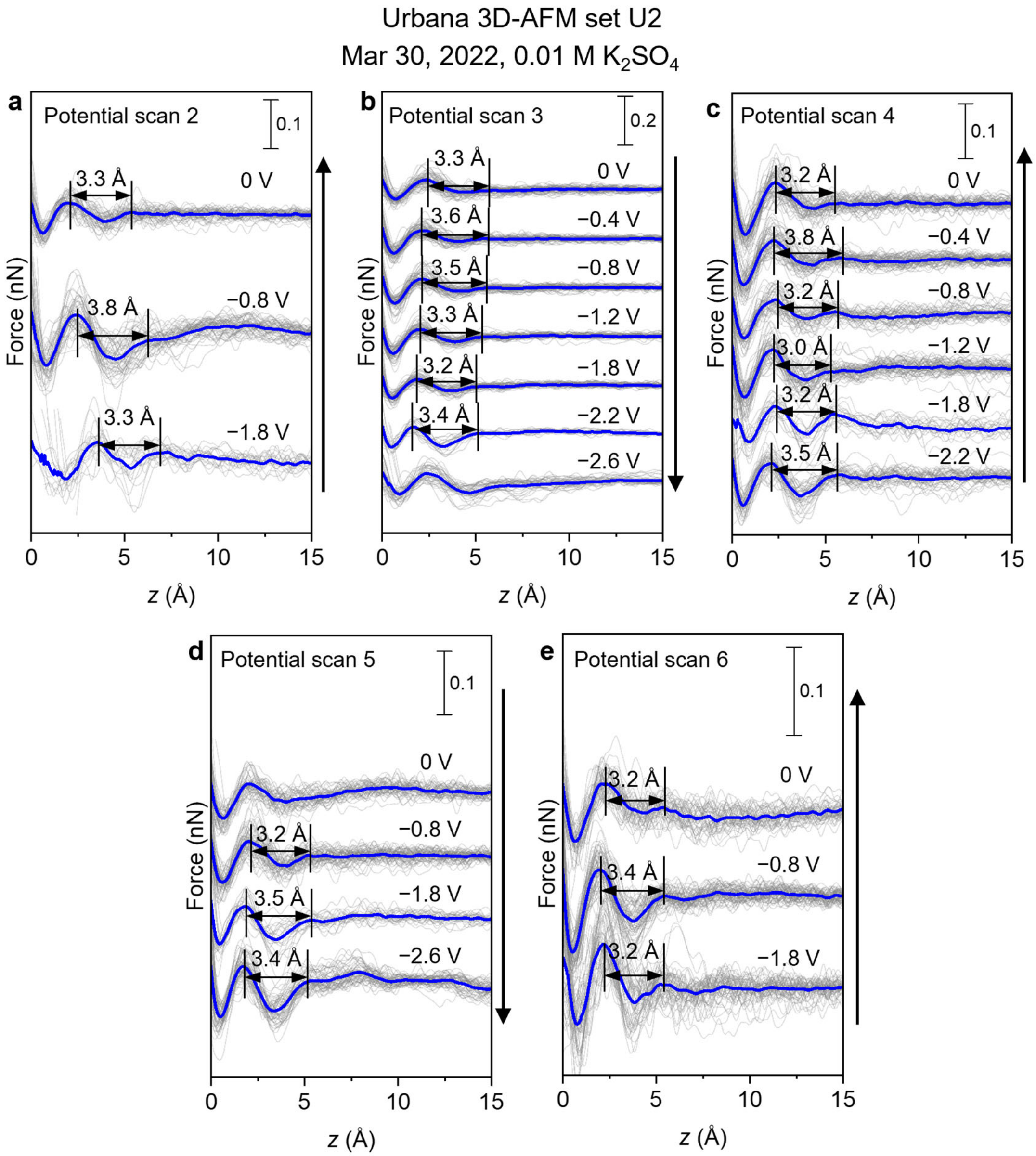

Supplementary Fig. 6 | Urbana 3D-AFM data set U2. Sample: HOPG/0.01 M K_2SO_4 solution. Gas environment: argon, sealed. The pristine state in (**a**) was achieved after a first electrochemical cleaning scan as described in Supplementary Fig. 12a. Throughout the five potential scans shown in (**a**–**e**), d_{12} was observed to mostly remain within 3–3.5 Å, corresponding to a pristine, water-dominant interface. Source data are provided as a Source Data file.

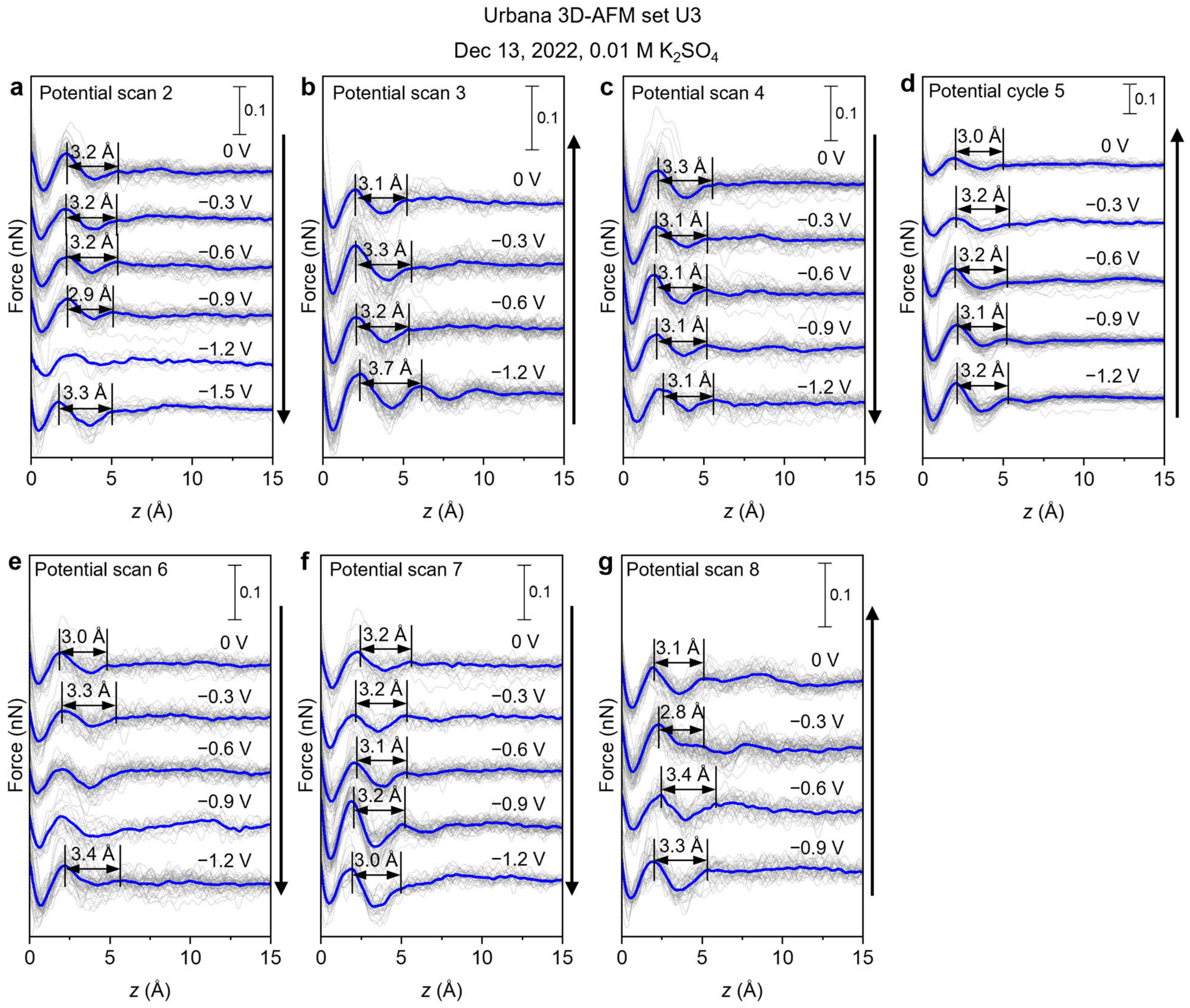

Supplementary Fig. 7 | Urbana 3D-AFM data set U3. Sample: HOPG/0.01 M K_2SO_4 solution. Gas environment: argon, sealed. Before the measurement shown in (**a**), a pristine state was achieved by applying negative electrode potentials until d_{12}=3.3 Å was observed, as shown in Supplementary Fig. 12b. Throughout all the seven scans shown here, d_{12} remained mostly constant near 3 Å, indicating that the pristine, water-dominant interface was retained. Source data are provided as a Source Data file.

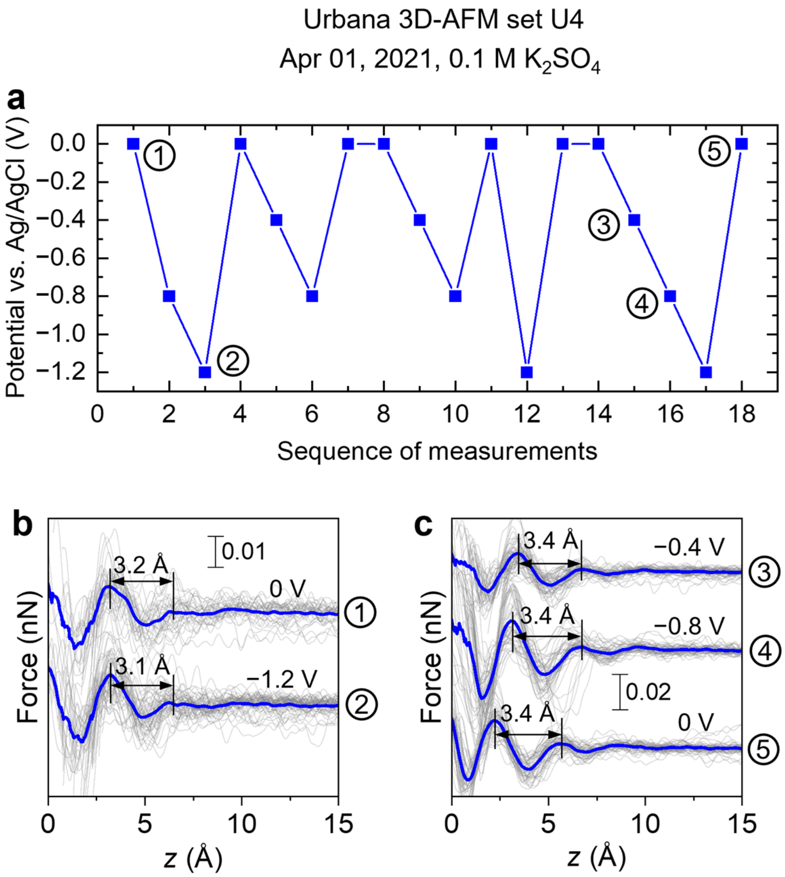

Supplementary Fig. 8 | Urbana 3D-AFM data set U4. Sample: HOPG/0.1 M K_2SO_4 solution. Gas environment: argon, sealed. **a**, Sequence of applied electrode potentials. **b**,**c**, Force–distance curves taken from specific potential points marked 1–5 in (**a**). A nearly constant d_{12} near 3 Å was observed in all the measured curves. Source data are provided as a Source Data file.

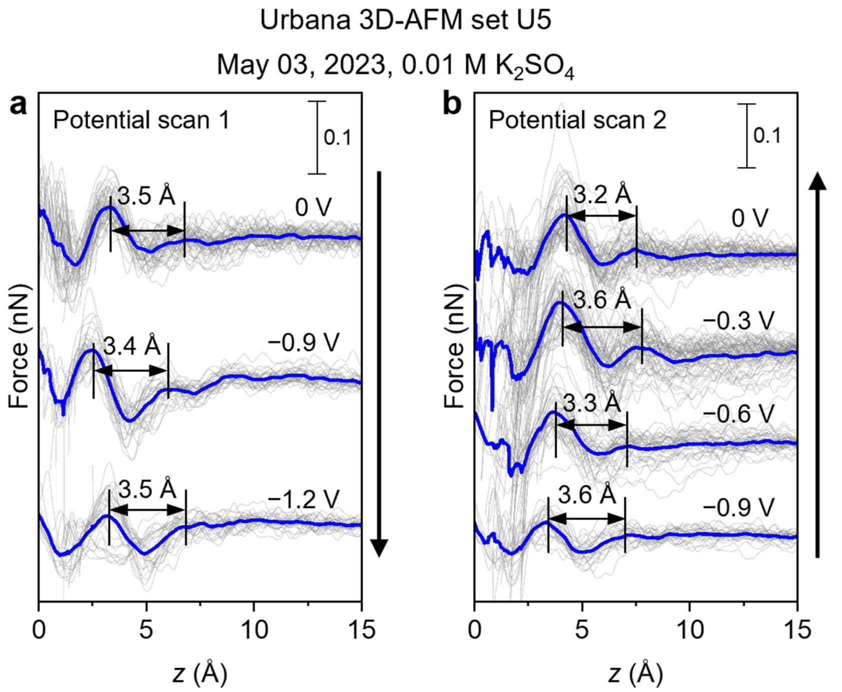

Supplementary Fig. 9 | Urbana 3D-AFM data set U5. Sample: HOPG/0.01 M K_2SO_4 solution. Gas environment: argon, sealed. The observed d_{12} remained within 3.2–3.6 Å throughout the two potential scans, indicating a mostly pristine interfacial state. Source data are provided as a Source Data file.

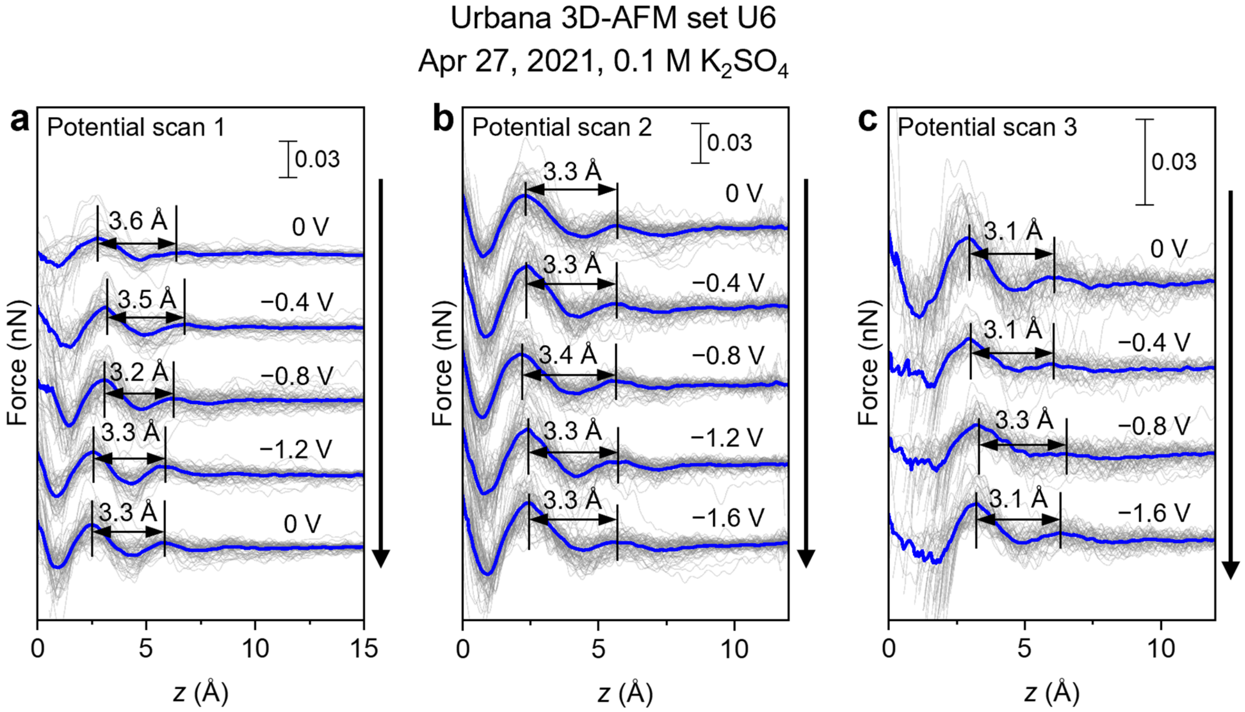

Supplementary Fig. 10 | Urbana 3D-AFM data set U6. Sample: HOPG/0.1 M K_2SO_4 solution. Gas environment: argon, sealed. The observed d_{12} remained mostly within 3.1–3.4 Å, indicating a pristine state and the potential-independence of interlayer spacing. Source data are provided as a Source Data file.

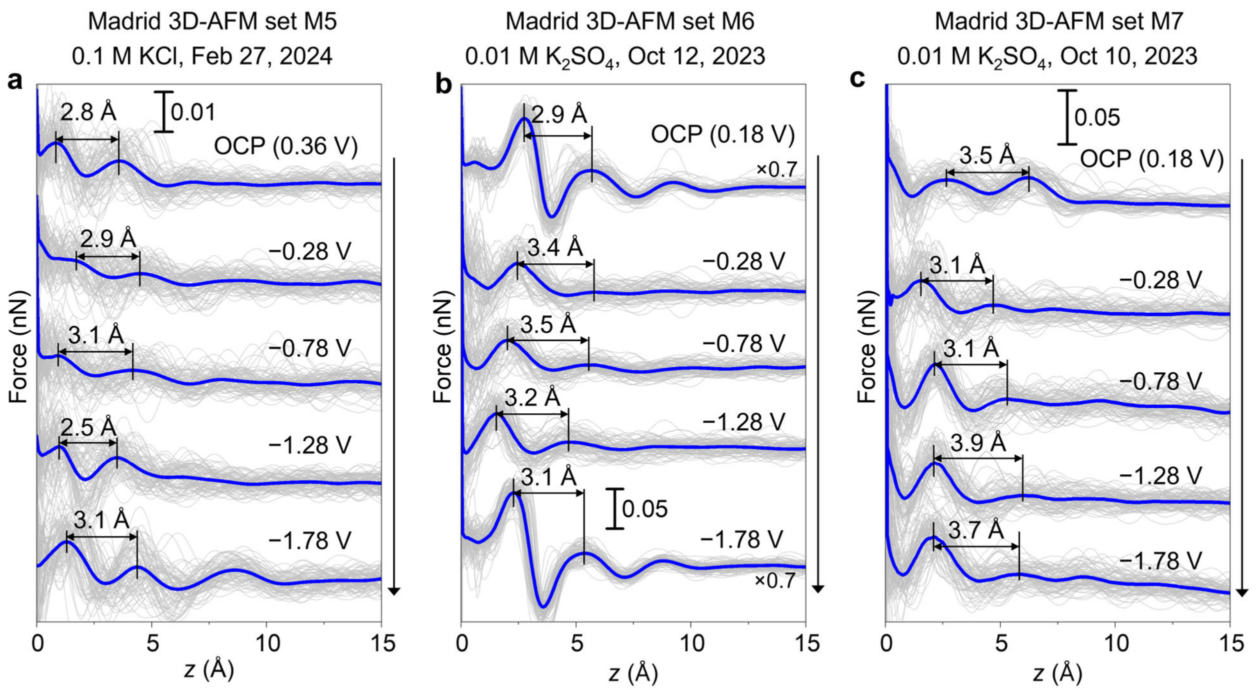

Supplementary Fig. 11 | Madrid 3D-AFM data sets M5–M7. Samples: HOPG/0.1 M KCl aqueous solution (**a**) and HOPG/0.01 M K_2SO_4 solution (**b**,**c**). Gas environment: air, open. In each measurement, an initial pristine interface was achieved by limiting the air exposure time to a few minutes before the first 3D-AFM map was obtained at OCP. Each data set with a complete potential scan was completed within a total time of ~20 minutes to minimize ageing effects. In all the data sets, d_{12} was consistently observed to be around 3 Å, regardless of the electrolyte composition/composition and electrode potential. Source data are provided as a Source Data file.

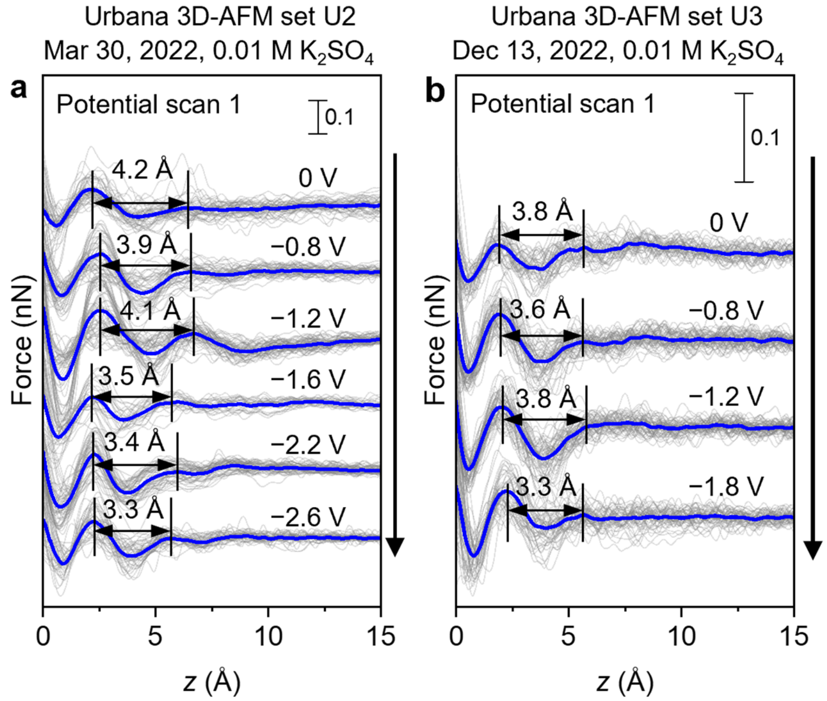

Supplementary Fig. 12 | Potential-dependent 3D-AFM measurements of an initially non-pristine HOPG/0.01 M K_2SO_4 solution interface. Data were collected at Urbana in an argon-sealed cell. The initial non-pristine state was evident from the observation of d_{12}≈4 Å from the initial 0 V data. The non-pristine states likely resulted from brief air exposures of the sample or the liquid cell during the initial preparation of the 3D-AFM imaging setup, before sealing the liquid cell with argon. In both data sets, d_{12} decreased from ≈4 Å to ≈3.3 Å at sufficiently negative potentials, indicating a transition from hydrocarbon-dominant to water-dominant interface. After the first potential scan, the following scans, shown in Supplementary Figs. 6 and 7, revealed a constant d_{12}≈3.3 Å, indicating that the pristine water-dominant states were retained. Source data are provided as a Source Data file.

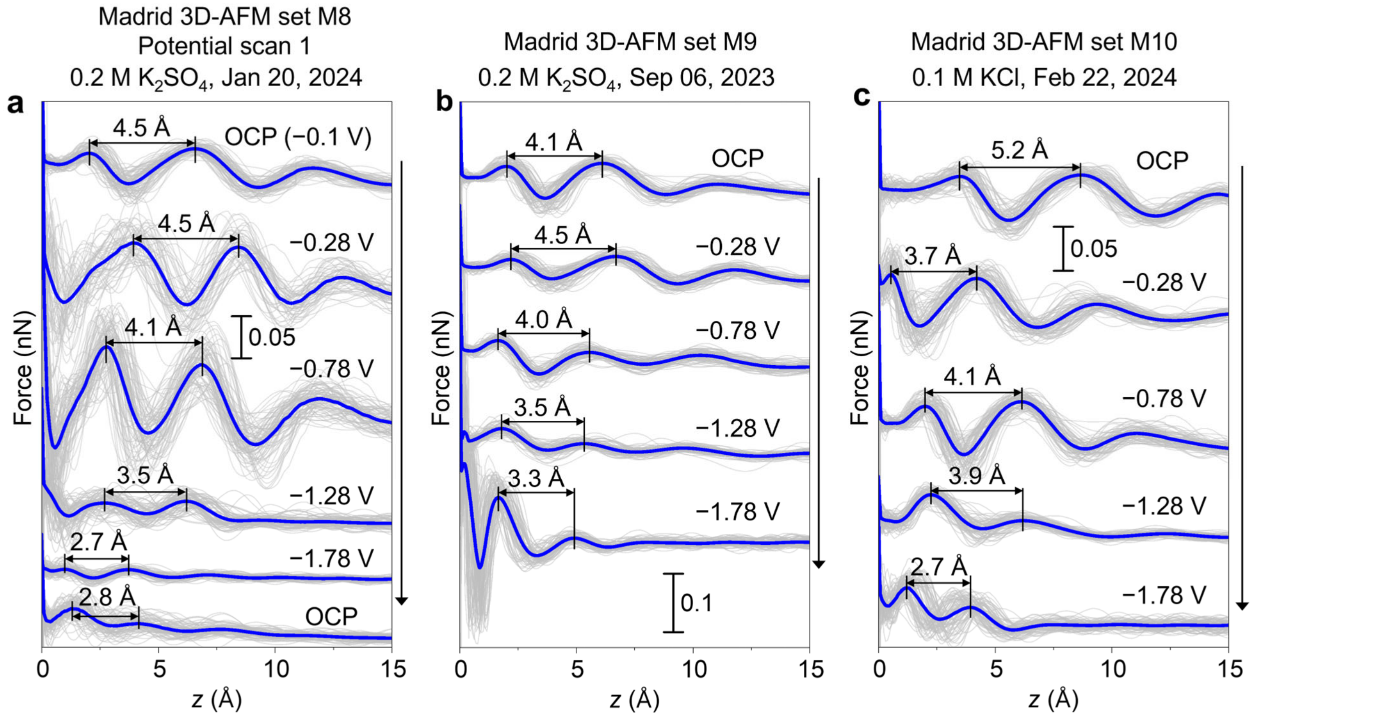

Supplementary Fig. 13 | Potential-dependent 3D-AFM of initially non-pristine HOPG/aqueous solution interfaces measured at Madrid. Samples: HOPG/0.2 M K_2SO_4 in water (**a**,**b**) and HOPG/0.1 M KCl (**c**). Gas environment: air, open. To induce the initial non-pristine states, the graphite surface with aqueous solution was left in ambient conditions for one hour. In all three measurement sets, the interlayer distance decreased from ≈4–5 Å to ≈3 Å at sufficiently negative potential, indicating a transition from hydrocarbon-dominant to water-dominant interface. The interlayer spacing remained at ≈2.8 Å right after electrode potential removal (see the post-cycling OCP curve in (**a**)). Following the measurements in (**a**), further 3D-AFM measurements were conducted at OCP to monitor the ageing process over time (see Supplementary Fig. 14b). The post-cycling curve in (**a**) measured at OCP is the same as corresponding one (0 min) in Fig. 2b (0.2 M K_2SO_4 panel) of the main article. Source data are provided as a Source Data file.

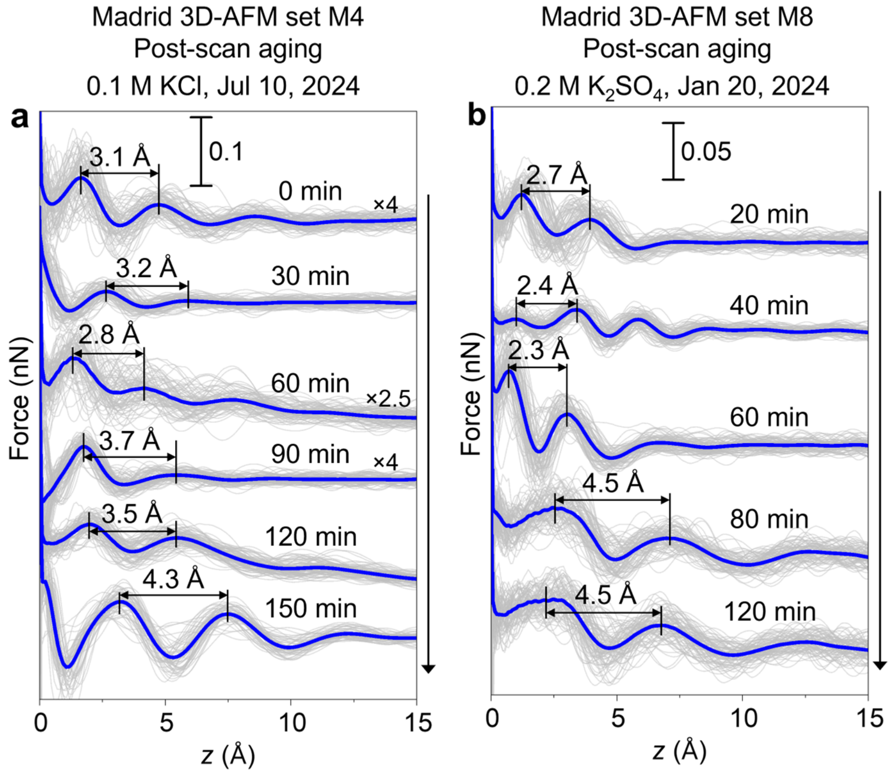

Supplementary Fig. 14 | Time evolution of force–distance curves after electrochemical cleaning of HOPG/aqueous solution interfaces. Data were acquired at Madrid in an open cell exposed to air. Samples: HOPG/0.1 M KCl aqueous solution (**a**) and HOPG/0.2 M K_2SO_4 aqueous solution (**b**). In both measurements, the sample was initially aged at OCP in ambient conditions for approximately one hour, followed by applying negative electrode potentials until a ≈3 Å interlayer distance was achieved. 3D-AFM measurements were subsequently performed at OCP at a series of time points, with results shown in this figure. For (**a**), 3D-AFM results were also recorded during both the initial aging (Supplementary Fig. 3d) and the following potential scan (Fig. 4a). For (**b**), 3D-AFM was not conducted during the initial ageing, but acquired in the following potential scan (see Supplementary Fig. 13a). In both data sets (**a**) and (**b**), the interlayer distance increased from ≈3 Å to ≈4.5 Å after more than 1 hour, indicating a transition from the pristine water-dominant state to the non-pristine hydrocarbon-dominant state. Note that the curves in (**b**) measured at 80 min are the same as corresponding ones (80 min) in Fig. 2b (0.2 M K_2SO_4 panel) of the main article. Source data are provided as a Source Data file.

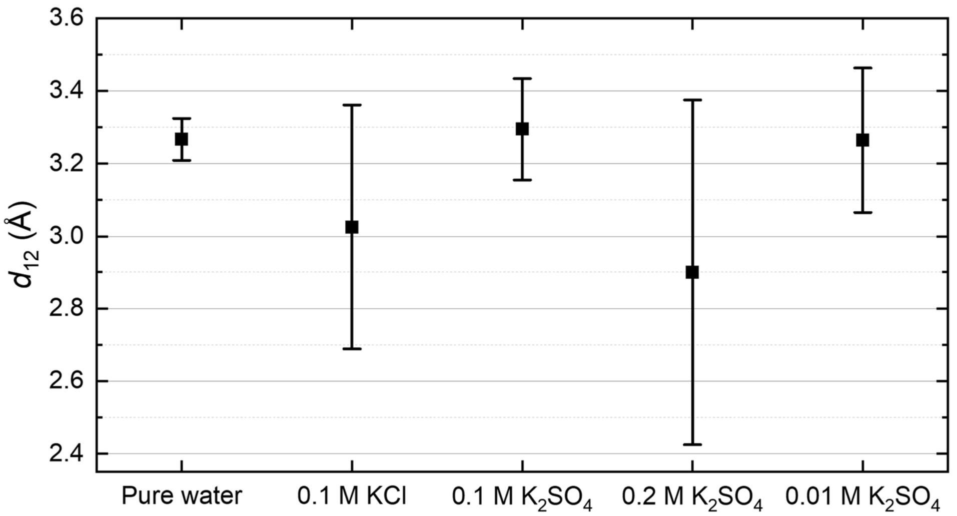

Supplementary Fig. 15 | Summary of interlayer distances (d_{12}) obtained from multiple 3D-AFM measurements at the pristine HOPG–aqueous solution interface across various electrolyte compositions (ion types and concentrations) and electrode potentials. Data are presented as mean ± SD calculated from technical replicates pooled across multiple independent experimental runs and electrode potentials (as specified below). Sample sizes (n) and the exact datasets are as follows: for pure water (n = 3), the 0-min data from datasets M1, M2, and M3 were used (corresponding to Supplementary Fig. 3a–c); for 0.1 M KCl (n = 12), M4 (0 min, post-scan aging at 0, 30, 60, 90, 120 min), M5, and M10 (−1.78 V) were used (Supplementary Figs. 3d, 14a, 11a, and 13c); for 0.1 M K_2SO_4 (n = 19), datasets U4 and U6 were used (Supplementary Figs. 8 and 10); for 0.2 M K_2SO_4 (n = 8), datasets M8 (−1.28 V, −1.78 V, and post-scan aging at 20, 40, 60 min) and M9 (−1.28 V, −1.78 V) were used (Supplementary Figs. 13a,b and 14b); for 0.01 M K_2SO_4 (n = 107), datasets U1, U2 (including potential scan 1: −1.6 V, −2.2 V, −2.6 V), U3 (including potential scan 1: −1.8 V), U5, M6, and M7 were used (Supplementary Figs. 5–7, 9, 11b,c, and 12a,b). Source data are provided as a Source Data file.

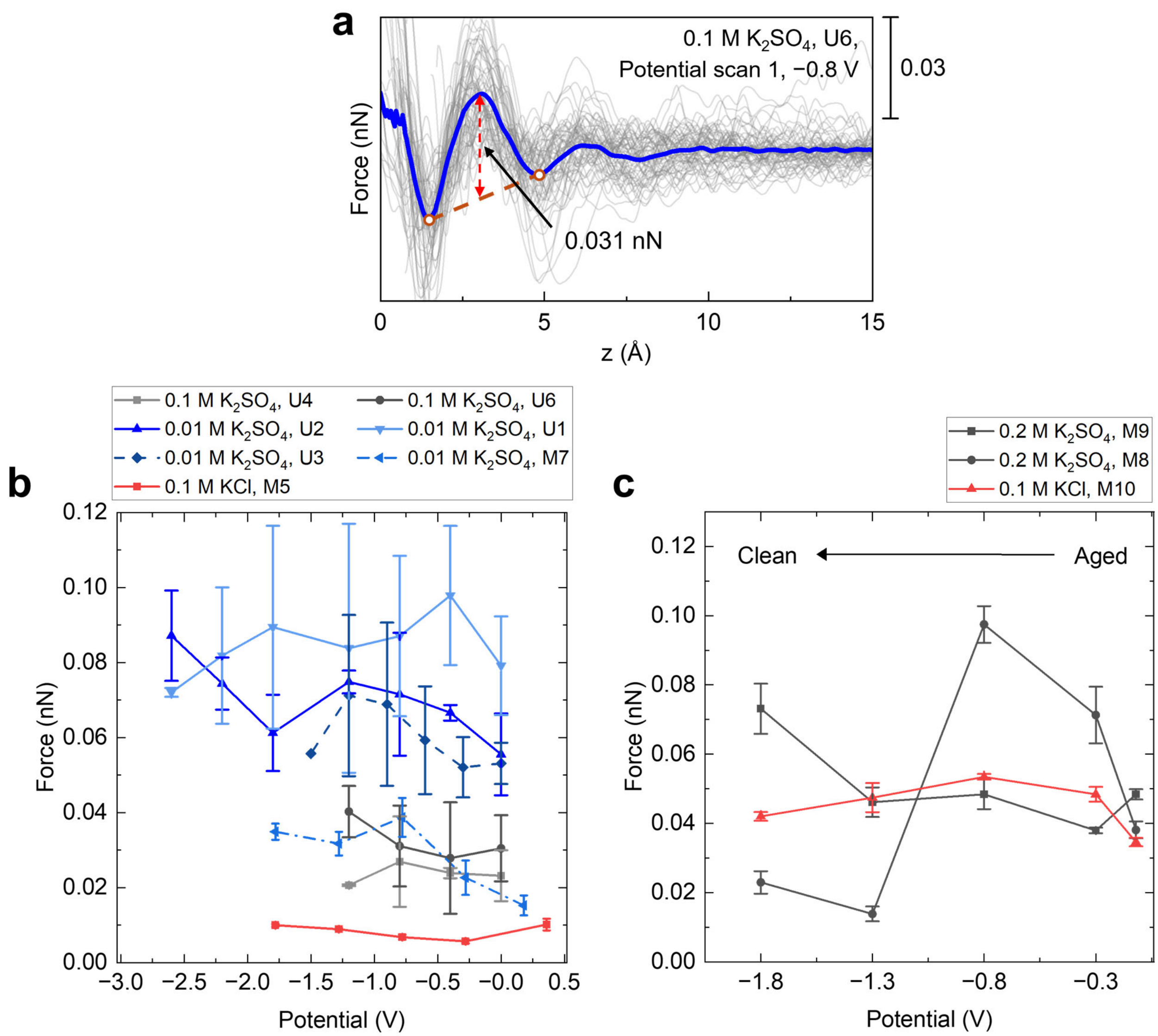

Supplementary Fig. 16 | Force oscillation amplitude analysis of 3D-AFM datasets. a, A representative 3D-AFM dataset (corresponding to Supplementary Fig. 10a, −0.8 V) together with schematics depicting the extraction of the force oscillation amplitude. **b**,**c**, Summary of measured force oscillation amplitudes from representative 3D-AFM datasets as a function of electrode potential for (**b**) pristine HOPG–electrolyte interfaces (corresponding to Supplementary Figs. 5–10, 11a, and 11c) and (**c**) non-pristine interfaces (corresponding to Supplementary Fig. 13) with different electrolyte compositions and concentrations. The overall spread in force values exceeds any systematic dependence on electrolyte concentration or ion identity. Data are presented as mean ± SD obtained from technical replicates pooled from individual 3D-AFM experiments. Sample sizes (n) for U1 (4, 5, 5, 5, 4, 5, 2), U2 (5, 2, 5, 2, 5, 2, 3), U3 (5, 6, 6, 5, 5, 1), U4 (9, 3, 3, 2) and U6 (5, 4, 4, 2) correspond to electrode potentials from 0 V to their respective negative limits. For all electrode potentials across M5, M9, and M10, $n = 80$. For M7, $n = 81$ for all potentials except for −1.78 V ($n = 80$). For M8, $n = 80$ for all potentials except for −0.28 V ($n = 54$) and for −1.28 V ($n = 81$). Source data are provided as a Source Data file.

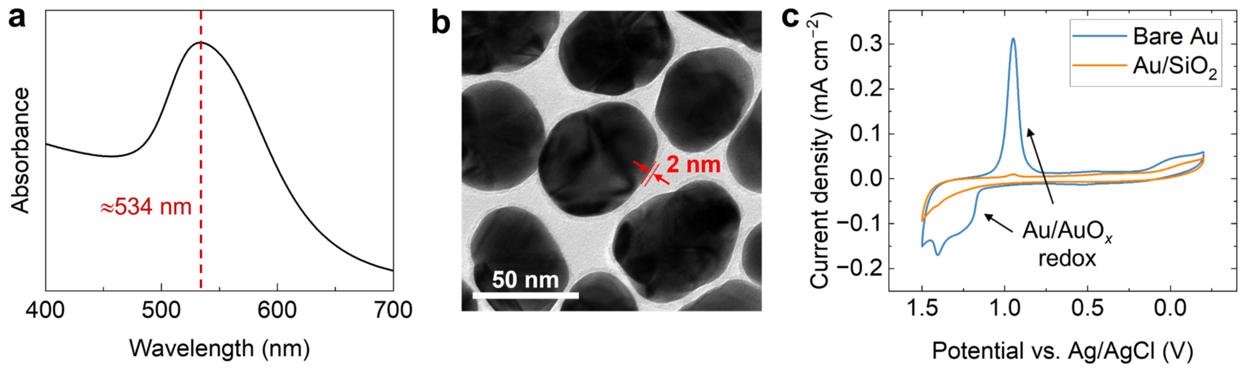

Supplementary Fig. 17 | Au/SiO_2 core/shell nanoparticle characterization. a, UV-Vis absorption spectrum of bare Au nanoparticles. According to Mie theory, the absorption peak at ≈534 nm corresponds to Au NPs with a diameter of 60 nm,[8,9] which is consistent with the diameter of 61.5±5.8 nm for the Au/SiO_2 core/shell NPs obtained from the scanning electron microscopy (SEM) image in Supplementary Fig. 18. The peak width of this spectrum is similar to previous reports of high quality Au NPs for SHINERS studies[10], further confirming the high uniformity of our nanoparticles. **b**, Transmission electron microscopy (TEM) image of Au/SiO_2 core/shell nanoparticles. The silica-shell thickness of ≈2 nm is marked. The image reveals the uniform coverage of the SiO_2 shell on the Au core. To acquire TEM images, the Au/SiO_2 particles were drop-casted onto a carbon film-coated copper TEM grid and then imaged using a JEOL 2100 TEM with a LaB_6 emitter at 200 kV. **c**, Cyclic voltammetry curves of bare Au and Au/SiO_2 particles deposited on glassy carbon electrodes in 0.5 M H_2SO_4 in water. Scan rate: 50 mV/s. The lack of Au/AuO_x redox peaks for the Au/SiO_2 particles further confirms the pinhole-free nature of the SiO_2 shell[10–12], consistent with TEM observations. Source data are provided as a Source Data file.

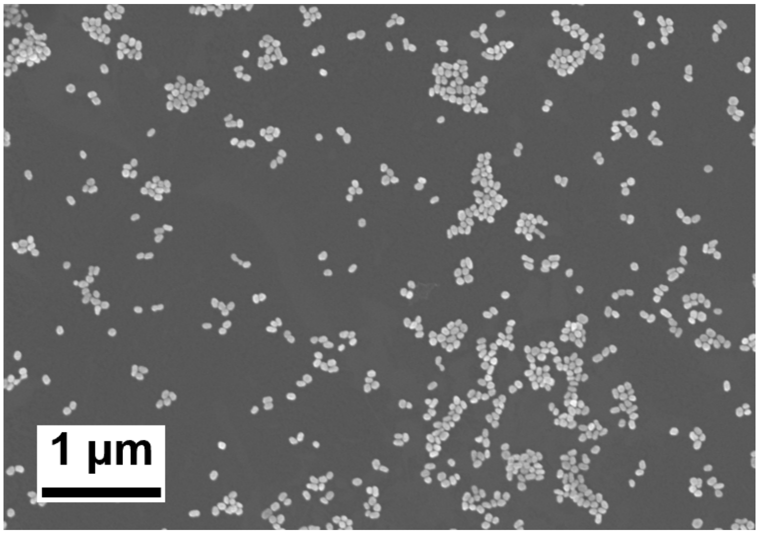

Supplementary Fig. 18 | Spatial distribution of Au/SiO_2 core/shell nanoparticles for SHINERS measurements. The SEM image was taken for Au/SiO_2 nanoparticles on HOPG at an approximate location where SHINERS measurements were conducted. The particle diameter is determined to be 61.5±5.8 nm based on this image.

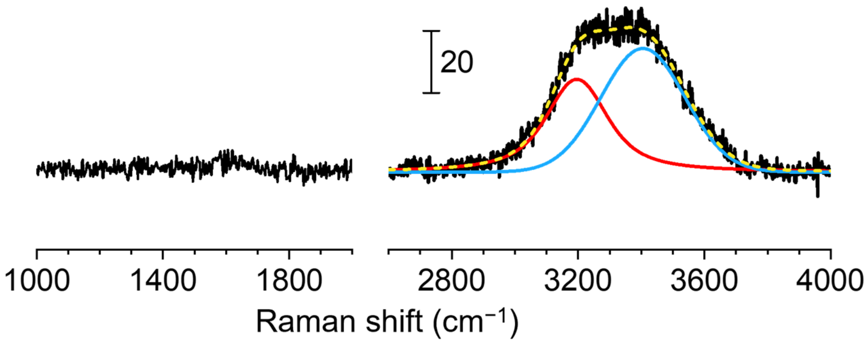

Supplementary Fig. 19 | Raman spectrum of the bulk 0.1 M K_2SO_4 aqueous solution. Gas environment: air, open. The ν_{OH} peak is deconvoluted into two Voigt peak components (red: OH-1, blue: OH-2) with their summed fit shown in as the yellow dashed line. The peak positions are 3196.03 cm^{-1} and 3405.31 cm^{-1} with integrated areas of 9735.58 counts cm^{-1} and 13277.3 counts cm^{-1}, respectively. The corresponding area fractions are 0.42 and 0.58. No peaks were observed in the FP region. All the SHINERS spectra of the HOPG/0.1 M K_2SO_4 in water interface in the ν_{OH} region were background-subtracted using this fitted bulk water spectrum, following the protocols illustrated in Supplementary Fig. 20. Source data are provided as a Source Data file.

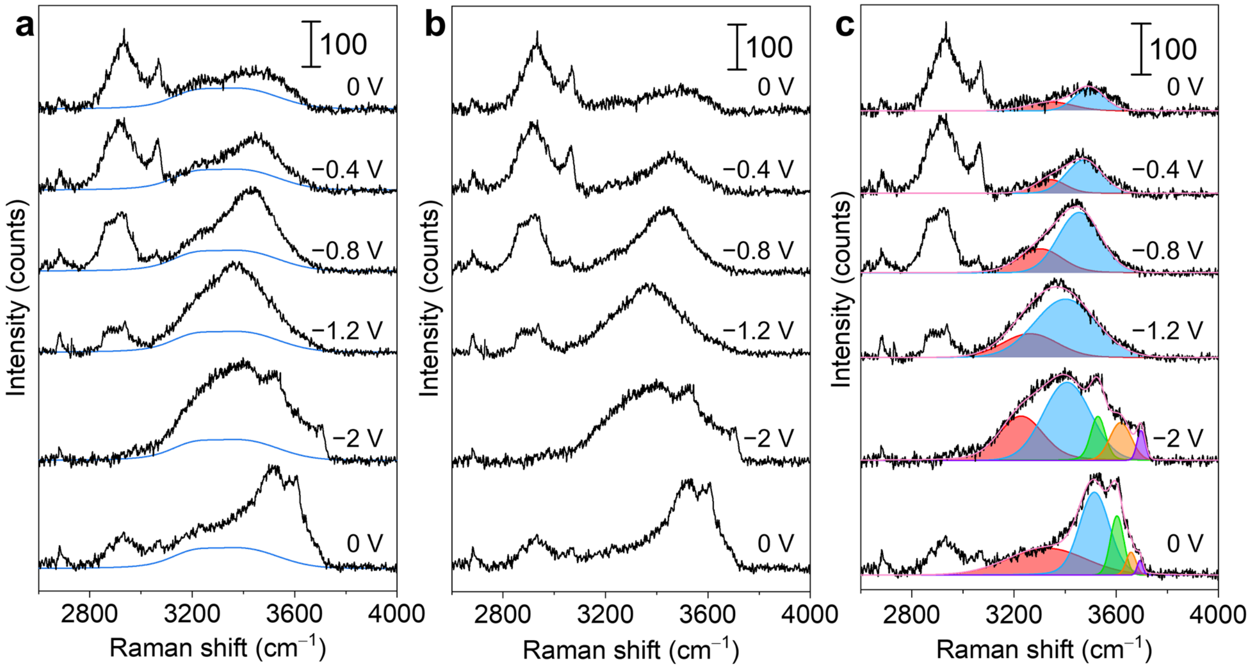

Supplementary Fig. 20 | SHINERS data processing steps. Results shown in Fig. 4c are chosen to illustrate the data processing protocols. **a**, Initial SHINERS spectra (black) along with the bulk aqueous solution spectrum (blue, same as the yellow dashed fit shown in Supplementary Fig. 19). **b**, SHINERS spectrum after removing bulk liquid contribution. **c**, Background-subtracted spectra (same as in (**b**)) along with Voigt fits of up to five ν_{OH} peak components and their sum.

Urbana SHINERS set 1
0.1 M K_2SO_4, Jun 25, 2023

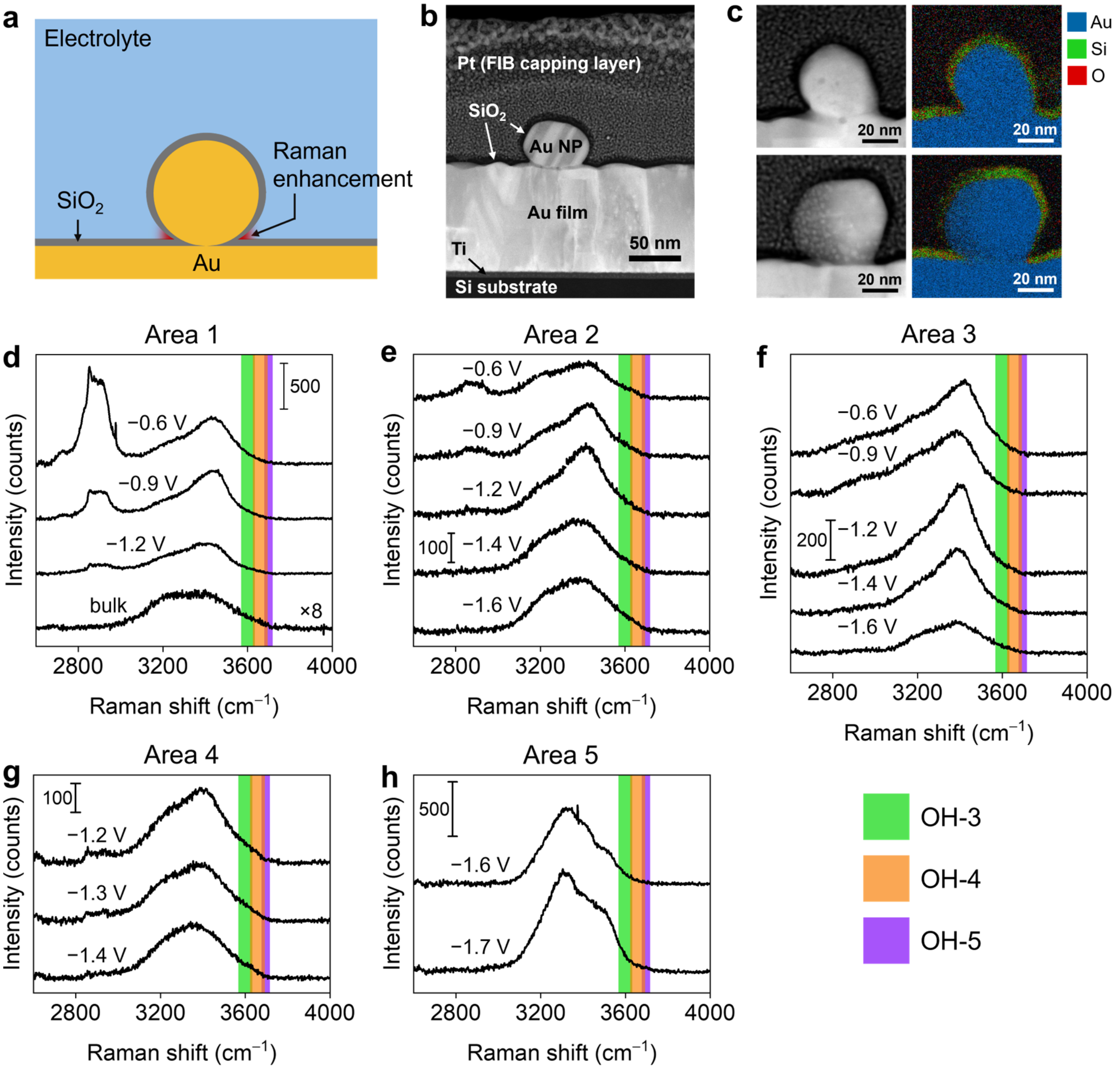

Supplementary Fig. 21 | SHINERS measurements of SiO_2/aqueous electrolyte interface. a, Schematic depicting the sample configuration, including the Au/SiO_2 substrate, Au nanoparticle with SiO_2 coating, and the liquid solution (0.1 M K_2SO_4 in water). The expected active region with the strongest Raman enhancement is also marked, which is the liquid electrolyte sandwiched between Au film/SiO_2 and SiO_2/Au nanoparticle. Briefly, the sample was prepared as follows: Using a silicon wafer served as the base substrate, 10 nm Ti / 100 nm thick gold layers were deposited via e-beam evaporation (using a Temescal e-beam evaporator). Then, bare Au nanoparticles were centrifuged, re-dispersed in Milli-Q water (following the same procedures used for the Au/SiO_2 particles) and subsequently drop-casted onto the substrate. After drying under argon, a ~3 nm-thick layer of silicon was sputter coated onto the particle-covered substrate (using an AJA Orion 3 sputter system). The silicon layer was then exposed to air, allowing oxidation to

form a silica layer. The fabricated substrate was then assembled into the electrochemical cell and sealed. An Ag/AgCl reference electrode (DEK Research Instrumentation, 3 M KCl) and a Pt coil counter electrode (DEK Research Instrumentation) were used for this measurement. **b**, Cross-section image of the substrate, obtained using high-angle annular dark-field scanning transmission electron microscopy (HAADF-STEM). A Pt capping layer was deposited before focused ion beam (FIB) cutting to prepare the cross-section for imaging. This Pt layer was absent in the Raman measurements. **c**, High resolution STEM images and energy-dispersive X-ray spectroscopy (EDS) elemental mapping of the cross-sectional substrate structure, confirming that a layer of SiO_2 was covering the Au nanoparticle/Au film substrate. **d**–**h**, SHINERS spectra obtained at a series of electrode potentials measured at multiple areas on the substrate. Gas environment: air, open. The wavenumber regions for OH-3, OH-4 and OH-5 (ν_{OH} components identified at the HOPG/solution interface) are marked by the green, orange, and violet rectangles, respectively. The center position and width of each of the rectangles are the same as the peak position and FWHM of the corresponding peak fits in Fig. 2c, ν_{OH} region of the 0 min panel. In all the spectra in (**d**–**h**), no discernable peaks can be identified in these regions expected for OH-3, OH-4 and OH-5. In (**d**), the bulk electrolyte Raman spectrum is plotted for comparison and is the same as the corresponding one shown in Supplementary Fig. 19. Note: for all the spectra presented in this figure, the contribution of bulk water was not subtracted. Source data are provided as a Source Data file.

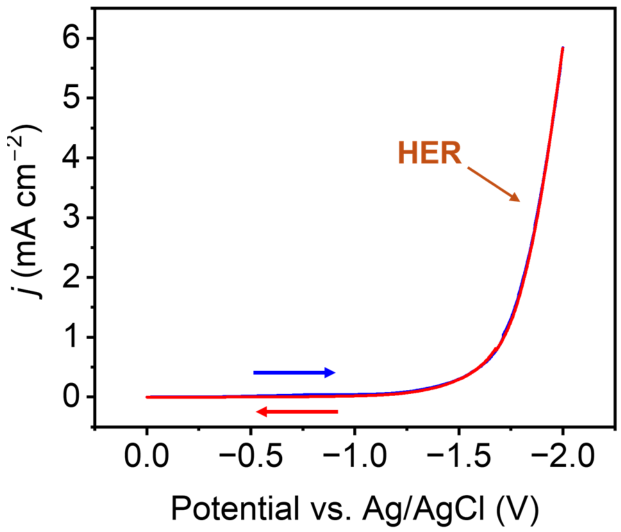

Supplementary Fig. 22 | Cyclic voltammetry of 0.1 M K_2SO_4 aqueous solution on HOPG. Scan rate: 5 mV/s. Electrode surface area: 1.44 cm^2. The red and blue arrows indicate the scan direction. The observed large current at potentials more negative than ≈−1.5 V is due to hydrogen evolution reaction (HER)[13]. No other redox reactions are observed. Source data are provided as a Source Data file.

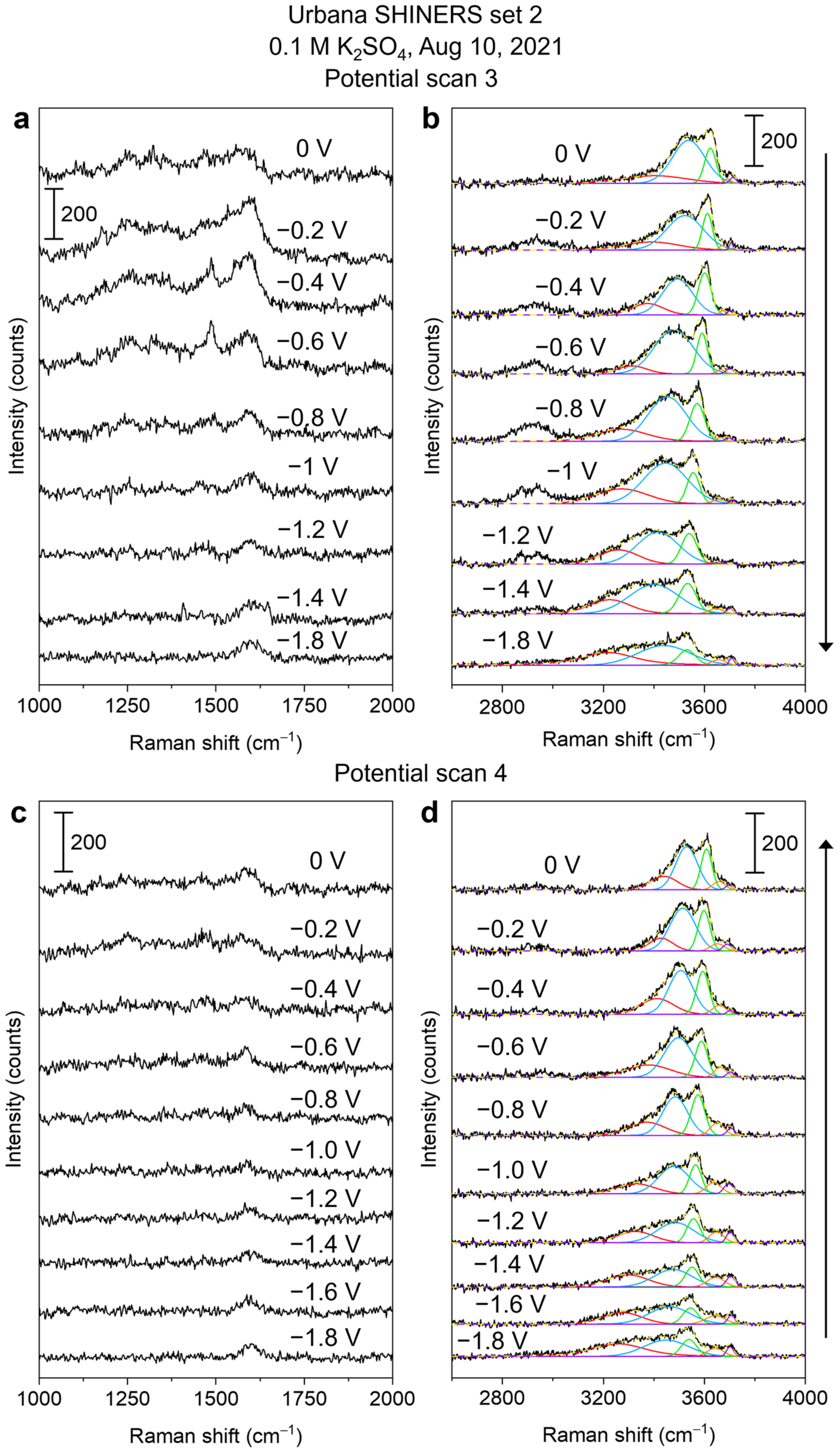

Supplementary Fig. 23 | Potential-dependent SHINERS of a pristine HOPG/0.1 M K_2SO_4 aqueous solution interface. Data were recorded on August 10, 2021 in Urbana. Gas environment: air, open. Reference electrode: Ag/AgCl. Arrows indicate the direction of potential changes. The pristine state was achieved by applying negative potentials as shown in Supplementary Fig. 26.

Negligible or weak FP or ν_{CH} peaks were observed in (**a**–**d**). ν_{OH} was deconvoluted into five components (OH-1 to OH-5) represented by red, blue, green, orange and violet curves, respectively. Yellow dashed lines correspond to the total fit. Note that the spectra in (**c**,**d**) are the same as the corresponding ones in Fig. 3c of the main article. Source data are provided as a Source Data file.

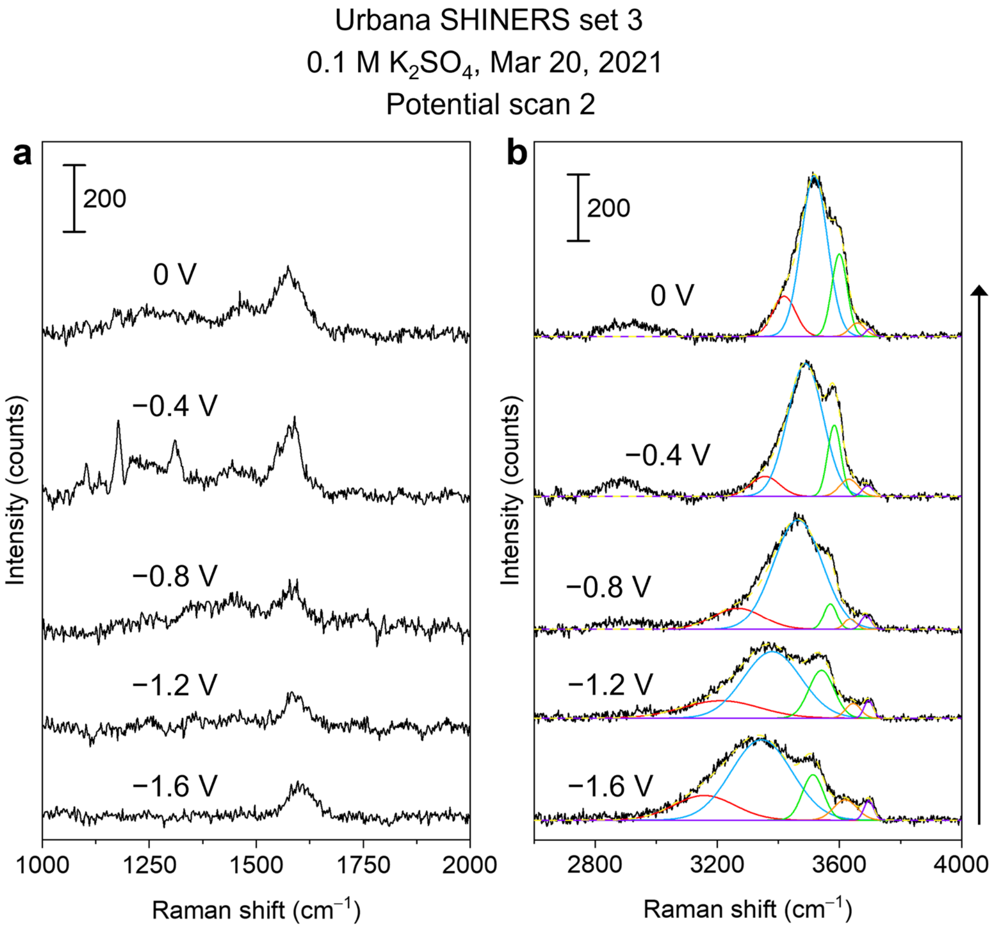

Supplementary Fig. 24 | Potential-dependent SHINERS at a pristine HOPG/0.1 M K_2SO_4 aqueous solution interface (Urbana SHINERS data set 3). Gas environment: air, open. Reference electrode: Ag/AgCl. Arrow indicates the direction of potential change. To achieve the pristine state, the electrode was subjected to a negative potential scan, shown in Supplementary Fig. 27, before acquiring the data shown here. ν_{OH} was deconvoluted into five peaks, OH-1 to OH-5, shown as solid colored curves, with increasing peak position. The yellow dashed line represents the summed fit of all components. The spectra at 0 V in (**a**,**b**) are the same as Fig. 2c, 0 min panel in the main article. Source data are provided as a Source Data file.

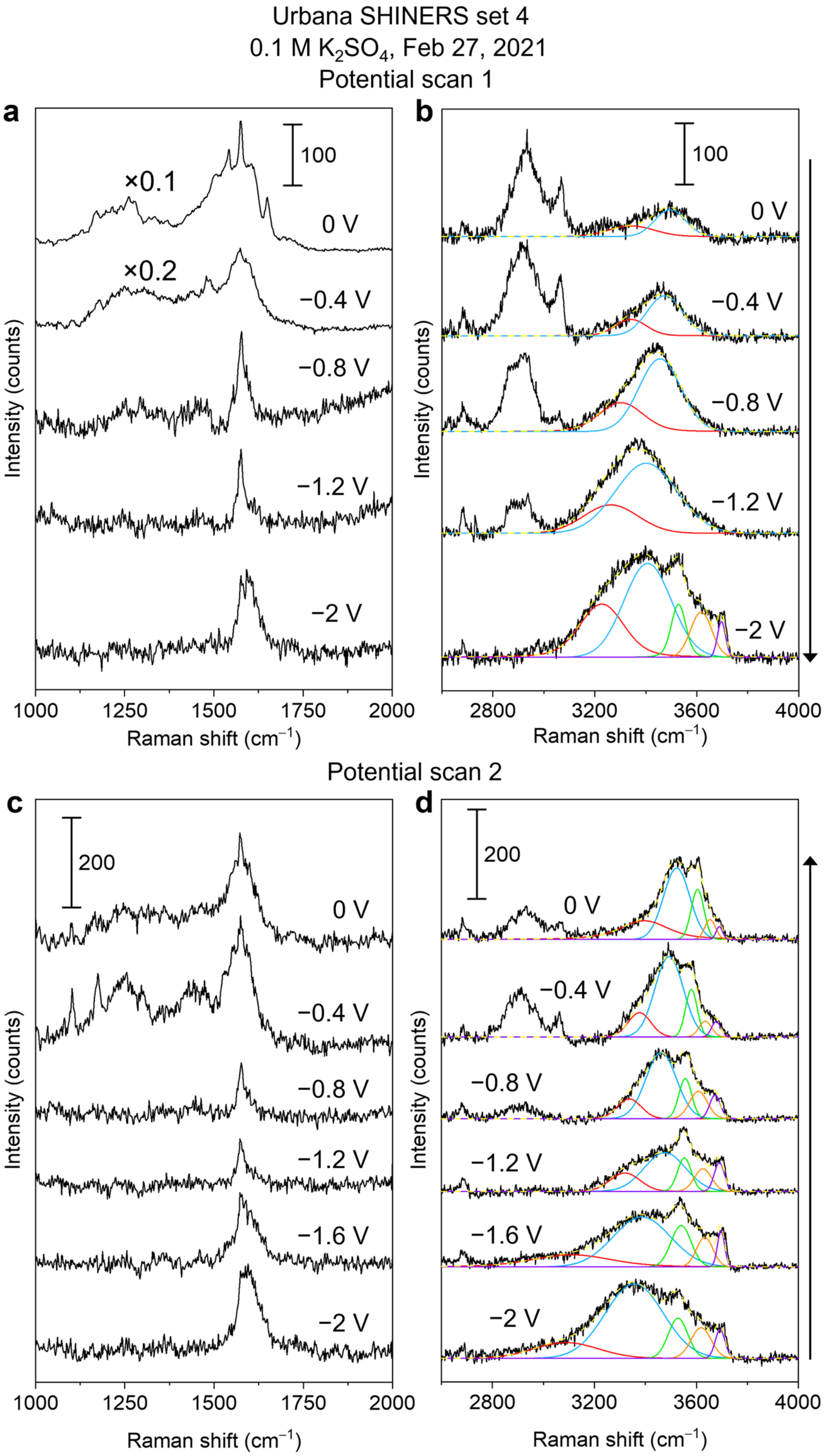

Supplementary Fig. 25 | Potential-dependent SHINERS of an initially non-pristine HOPG/0.1 M K_2SO_4 aqueous solution interface. These measurements were conducted on Feb 27, 2021 (Urbana) using an open cell exposed to air. Reference electrode: Ag/AgCl. Arrows indicate the sequence of applied potentials. Note that the results in (**a**,**b**) and the 0 V data in (**c**,**d**) are the same as the corresponding ones in Fig. 4c of the main article. Source data are provided as a Source Data file.

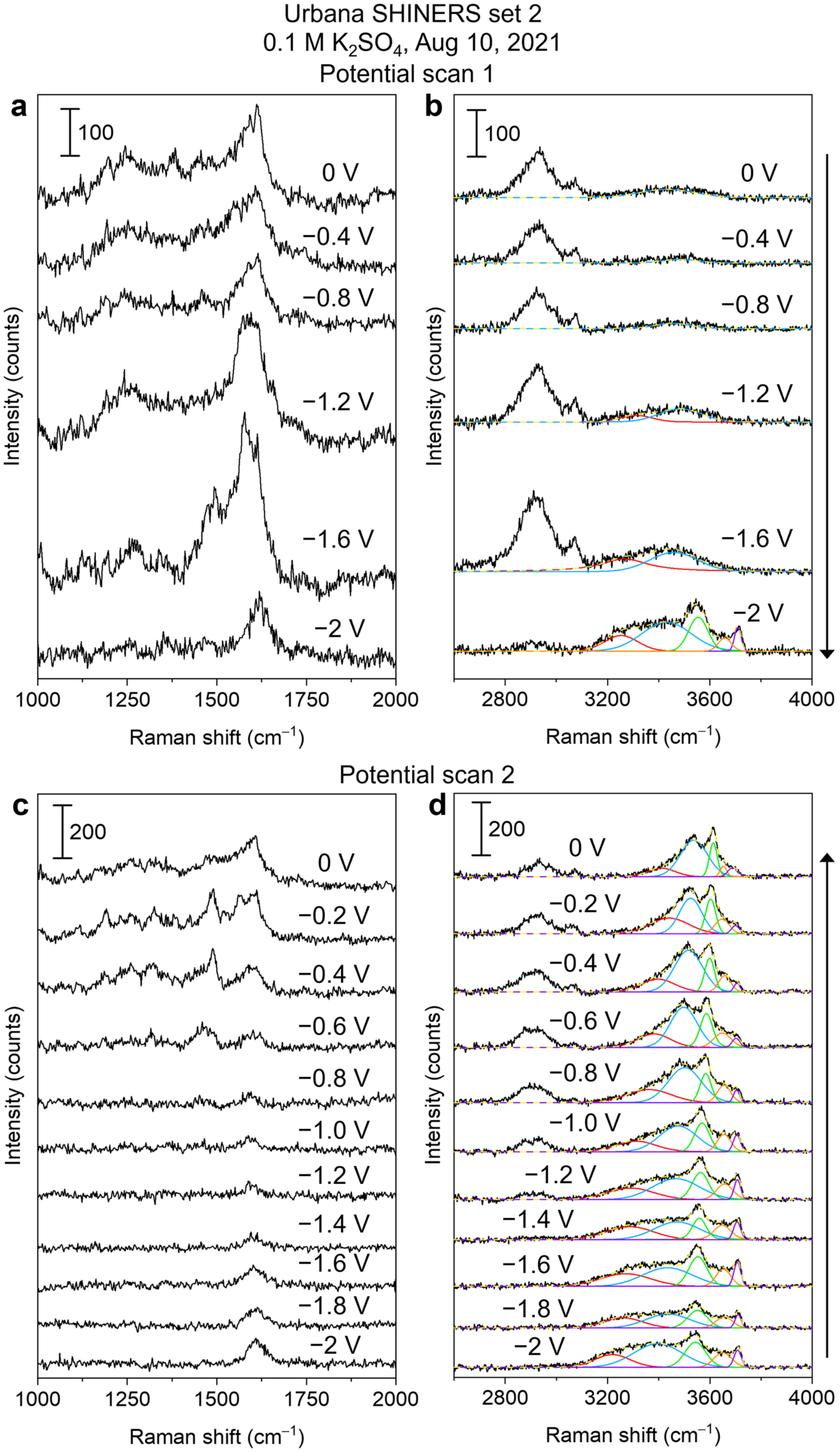

Supplementary Fig. 26 | Potential-dependent SHINERS of an initially non-pristine HOPG/0.1 M K_2SO_4 aqueous solution interface (Urbana data set 2). These measurements were conducted using an open cell exposed to air. Reference electrode: Ag/AgCl. Source data are provided as a Source Data file.

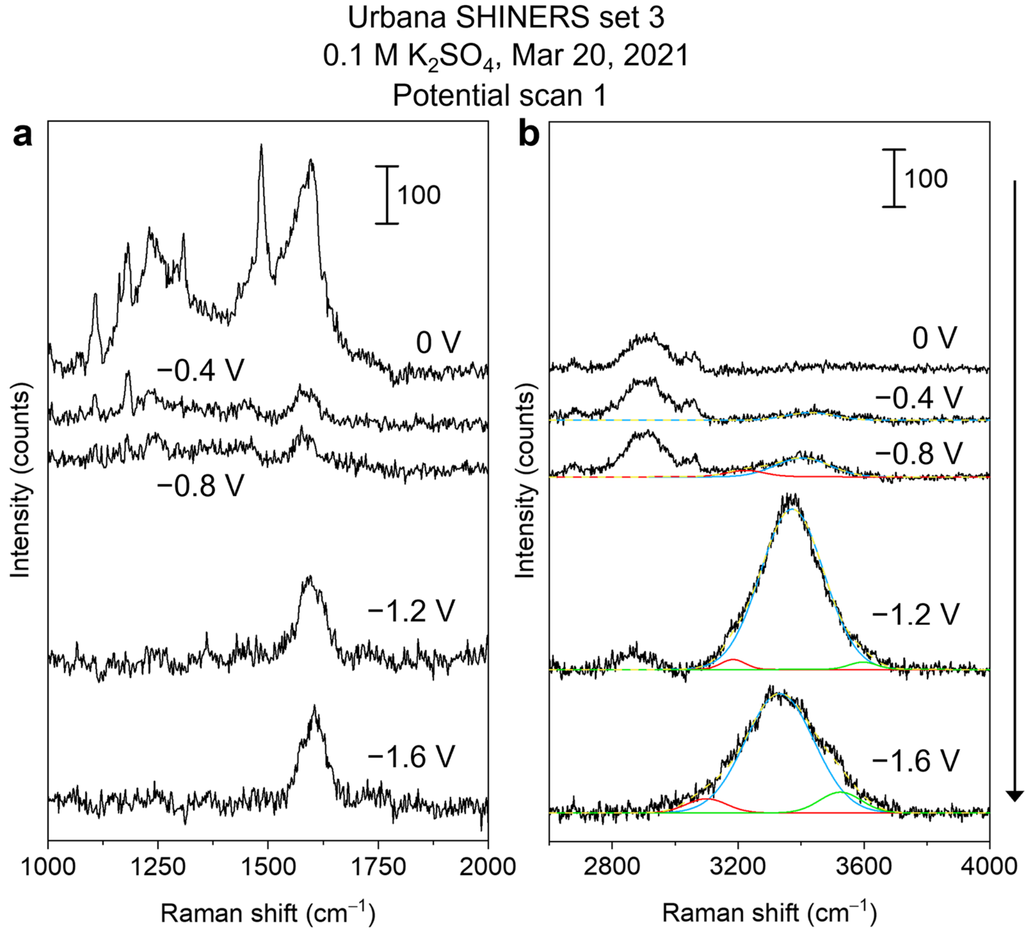

Supplementary Fig. 27 | Potential-dependent SHINERS of an initially non-pristine HOPG/0.1 M K_2SO_4 aqueous solution interface (Urbana data set 3). Gas environment: air, open. Reference electrode: Ag/AgCl. Source data are provided as a Source Data file.

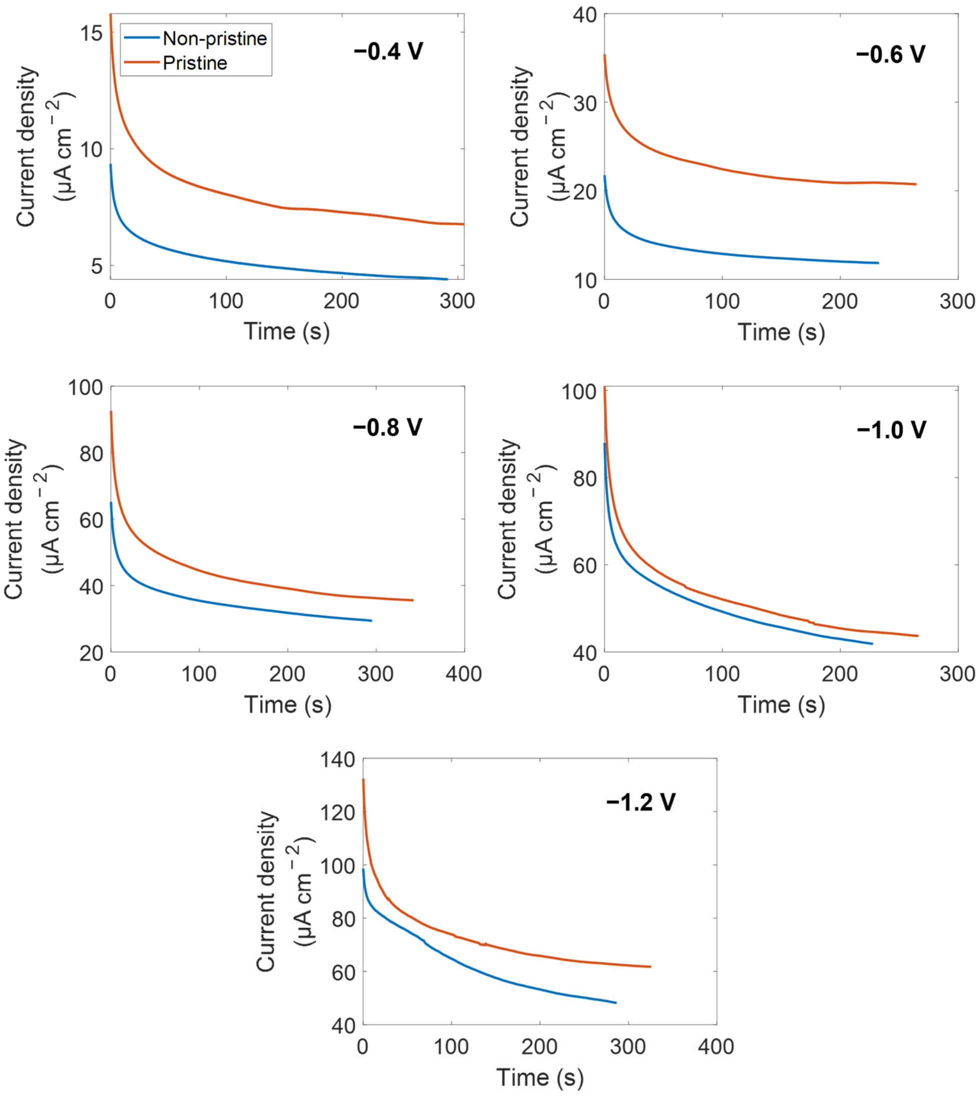

Supplementary Fig. 28 | Current transients recorded during SHINERS measurements (August 10, 2021, Urbana). Gas environment: air, open. Potential reference: Ag/AgCl. The pristine and non-pristine data were recorded during SHINERS measurements shown in Supplementary Fig. 23 (potential cycle 3) and Supplementary Fig. 26 (potential cycle 1), respectively. The electrode potential was changed at a step of –0.2 V, and was held constant (at the values specified on each panel) while the current transient was recorded. The pristine interface consistently exhibited larger current transients than those of the non-pristine one, revealing that the former has higher double layer capacitance than the latter. This is likely because the hydrocarbons accumulated at the HOPG–solution interface induce a larger spatial separation between the electrical double layers and the HOPG surface, which further leads to smaller capacitance. Overall, these observations suggest that the transient current may serve as a qualitative indicator of surface cleanliness. Source data are provided as a Source Data file.

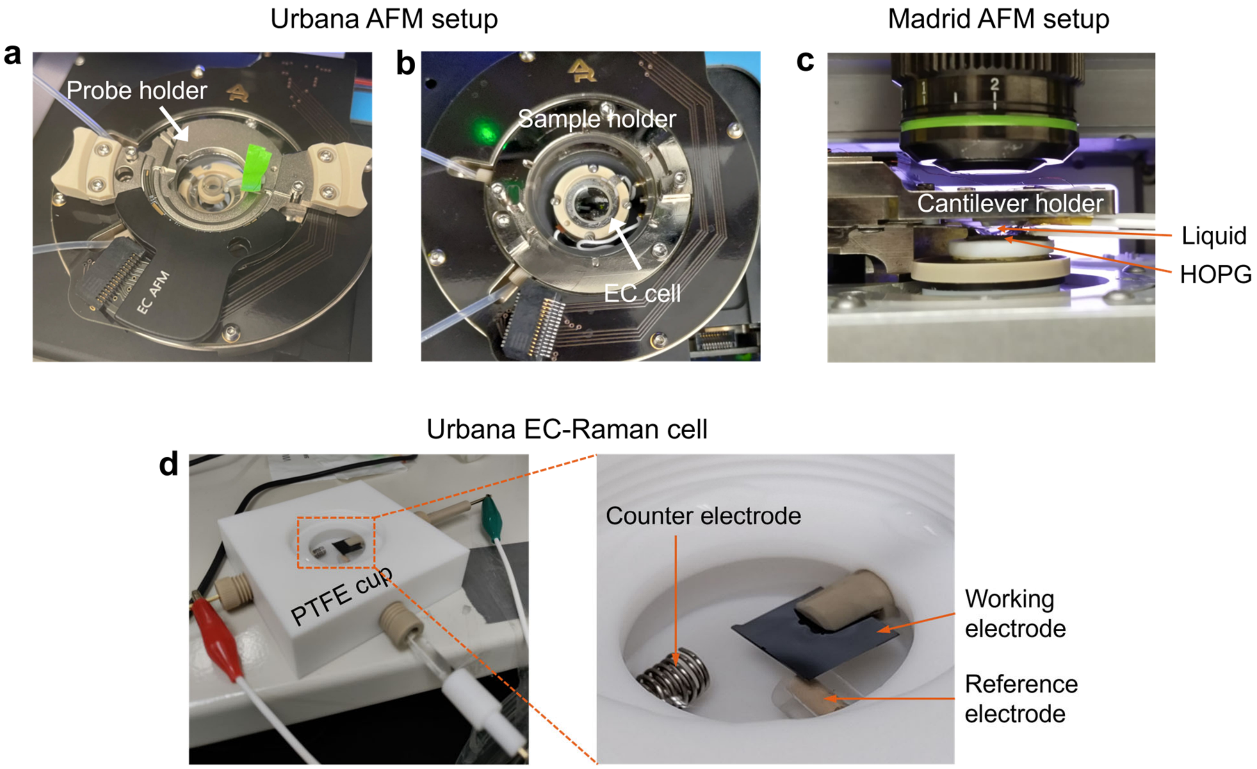

Supplementary Fig. 29 | Photos of key experimental setups. a,**b**, Urbana AFM setup in sealed condition (**a**) and with AFM probe holder removed showing the underlying electrochemical (EC) cell (**b**). **c**, Madrid open-cell AFM setup. **d**,**e**, Urbana EC-Raman cell configuration.

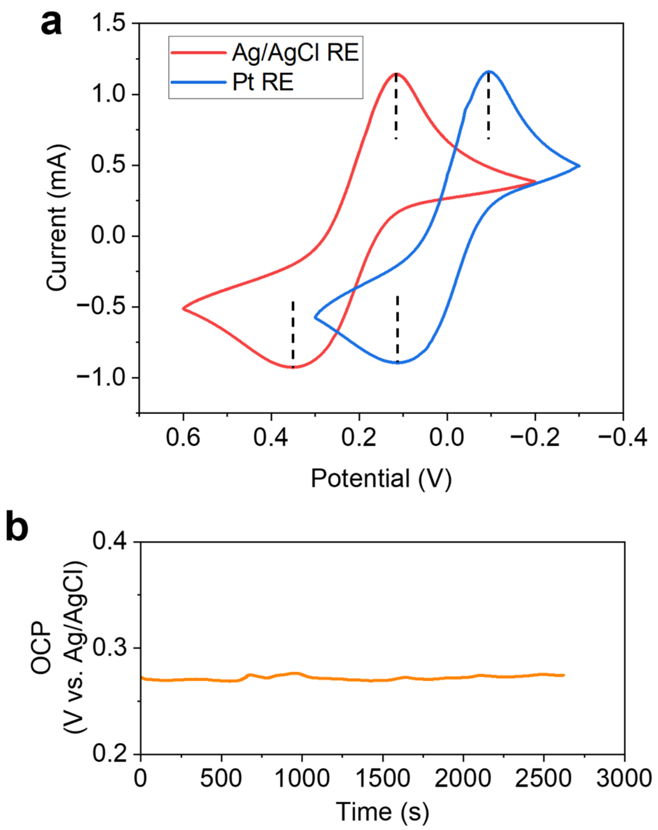

Supplementary Fig. 30 | Reference electrode calibration. a, Cyclic voltammetry curves of glassy carbon electrode / 0.1 M K_2SO_4 + 50 mM $K_4[Fe(CN)_6]$ + 50 mM $K_3[Fe(CN)_6]$ in water. Counter electrode: platinum. Reference electrode (RE): Ag/AgCl (shown in red) or platinum (shown in blue). Working electrode surface area: 19.64 mm^2. The potential of Pt was found to be 0.22 V more positive than Ag/AgCl. **b**, OCP recorded as a function of time for a platinum wire electrode versus an Ag/AgCl reference electrode in 0.1 M K_2SO_4 aqueous solution. The average OCP value was found to be ≈0.27 V. Source data are provided as a Source Data file.

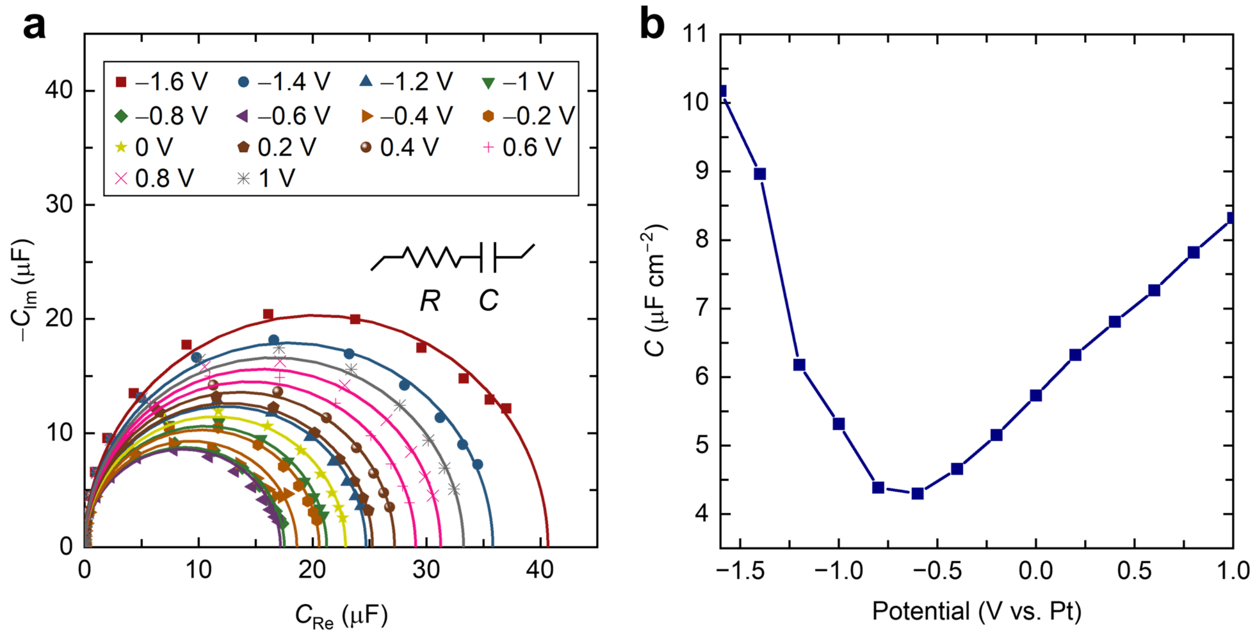

Supplementary Fig. 31 | Electrochemical impedance spectroscopy (EIS) measurements and capacitance analysis. Electrode: HOPG. Electrolyte: 0.1 M K_2SO_4 aqueous solution. **a**, EIS spectra at a series of potentials vs. Pt, plotted in complex capacitance plane. Measurements were performed using a Biologic potentiostat over the frequency range of 1 Hz to 1 MHz with an AC perturbation amplitude of 25 mV. The electrode surface area was 0.64 cm^2. Data in the range of 316 Hz to 1 MHz were used for fitting. Scattered dots correspond to the experimental data and lines are series RC circuit fits. **b**, Capacitance as a function of potential, extracted from RC circuit fits in (**a**). The capacitance vs. potential curve reveals a minimum at –0.6 V vs. Pt (–0.38 V vs. Ag/AgCl). Source data are provided as a Source Data file.

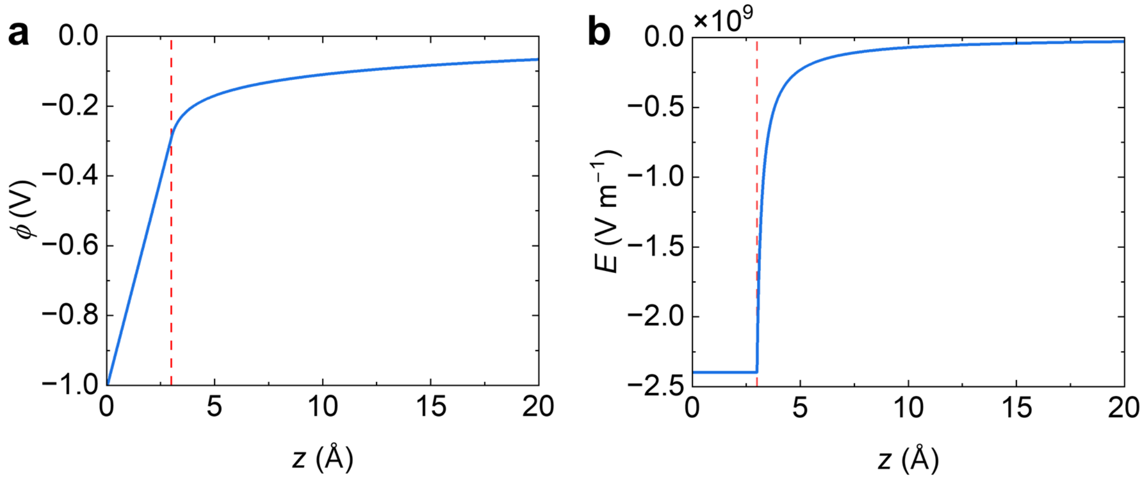

Supplementary Fig. 32 | GCS theory calculations. a, Potential (ϕ) and **b**, electric field (E) as a function of z. The red dashed line indicates the position of z_2 (3 Å from the electrode surface). Source data are provided as a Source Data file.

Supplementary Tables

Supplementary Table 1 | Summary of X-ray reflectivity (XRR) studies on solid–water interfaces

Solid substrate	Liquid	Inter-layer distance	Key conclusions	Ref.
Muscovite mica (001) (freshly cleaved, high-grade 2M_1 mica)	Water [ultrapure deionized water in equilibrium with atmosphere (pH ≈5.7)]	2.7 Å between 1st–2nd layer; 3.7 Å for upper layers	Pristine; 4 layers observed; first hydration layer contains two sub-peaks (1.3 Å and 2.5 Å above the substrate surface, respectively); 1st–2nd layer distance is 2.7 Å; upper layers are separated by 3.7 Å.	[14]
Epitaxial graphene [grown on 6H-SiC (0001) wafer via thermal decomposition in a vacuum oven]	Water (no specific source mentioned)	≈3 Å	Pristine; at least 2 layers observed; primary hydration layer is ≈3 Å from the surface; second layer at ≈6 Å.	[15]
Calcite (104) (freshly cleaved optical quality calcite)	Water [Nanopure (≈18 MΩ·cm) deionized water; pH ≈ 8.3]	≈3 Å	Pristine; at least 2 layers; primary layer at two separate heights (2.0 Å and 2.9 Å due to heterogeneous surface sites); second layer is ≈5 Å from the surface.	[16–18]
Rutile TiO_2 (110) (polished single crystals purchased from Princeton Scientific)	Water (ultra-filtered water, >18 MΩ·cm)	N/A	Pristine; one hydration layer identified at ≈2.1 Å from the surface.	[19]
Self-assembled monolayer (SAM) of octadecyltriethoxy siloxane (OTE) deposited on oxidized Si (100) (water contact angle > 100°)	Water (deionized; either deaerated or saturated with ambient gases)	N/A	An interfacial water depletion region with <40% electron density than bulk water. The thickness of the depletion region is ≈3 Å.	[20]
Paraffin (2D crystalline monolayer of the linear chain alkane n-$C_{36}H_{74}$)	Pure water	N/A	Density depletion layer: ≈15 Å thick; hydrophobic effect results in density deficit / dewetting; hydrophobic interfacial energy results in isothermal decompression of water.	[21]

Ag (111) (single crystals prepared using a chromic acid etch)	0.1 M NaF in water;<br>0.002 M NaF in water (ultrapure salt purchased from Johnson Matthey along with HPLC deionized water)	≈2–3 Å	Pristine; voltage-dependent; similar behavior was observed with two different concentrations; higher water accumulation in the first layer at positive potentials with closer proximity to the electrode; water reorientation and hydrogen-bond network disruption in the first layer.	[22,23]

Supplementary Table 2 | Summary of neutron reflectivity (NR) studies on solid–water interfaces

Solid substrate	Liquid	Inter-layer distance	Key conclusions	Ref.
SAMs of various hydrophobic and hydrophilic organic molecules. A single crystal SiO_2 (0001) coated with ~10 nm thick chromium and ~20–50 nm thick gold on top was used as a substrate for SAM deposition.	Deuterated water (D_2O) or D_2O/H_2O mixtures	N/A	≈4 nm depletion layer near the surface with ≈85–90% water compared to bulk water density; possible air accumulation or air nanobubble adsorption at hydrophobic interfaces; concerns were raised regarding the interpretations/reliability of the conclusions due to the limited quality of the substrate films (and possible air trapping in the films). For hydrophilic samples, either reduced or higher interfacial water density can fit the data.	24
SAM of octadecyl-trichlorosilane (OTS) attached to polished quartz substrates. Before each measurement, the substrates were cleaned with DI water, chloroform, ethanol and dried in ultra-high pure argon gas. (water contact angle > 100°)	Deuterated water (D_2O) saturated with Ar or CO_2, untreated (naturally aerated), or completely degassed	N/A	Gas-dependent depletion region, with a thickness of ≈10 Å for untreated D_2O, ≈2 Å for Ar-saturated D_2O, and ≈6 Å for CO_2-saturated D_2O. In the depletion region, the water density was <≈10% vs. the bulk water. Though gas dependence was observed, the observations were assumed to be unrelated to preexisting nanobubbles. The depletion region was tentatively attributed to fluctuating water density distributions at the interface (possibly related to fluctuating water vapors at the interface).	25
SAMs of deuterated dodecanethiol and hexadecanthiol	Pure water and aqueous solutions	N/A	Interfacial depletion of water; depletion is correlated with the affinity of the liquid to the solid surface.	26
Perdeuterated polystyrene spin-coated on silicon	Deuterated water (D_2O)	N/A	Depletion region: 2–5 nm thick; precursor gas layer occupies the interface, and the thickness depends on the level of air saturation and elapsed time.	27

Supplementary Table 3 | Summary of 3D-AFM studies on solid–water interfaces

Solid substrate	Liquid	Inter-layer distance	Key conclusions	Ref.
Graphite (HOPG, SPI-1 grade purchased from Alliance Biosystems, Japan)	Water (from water purifier, RFP742HA, Advantec, Japan); Just prior to the measurement, fresh HOPG was immersed in ethanol for several minutes and was exchanged slowly by injecting a large amount of pure water.	≈5–9 Å on bare clean surface; ≈7 Å on surface area with disordered topographic structures.	Mixed; different regions of surface were observed; gas layers were sandwiched between water layers, leading to larger interlayer distance of hydration layers.	[28]
Mica (mica plate from Ted Pella)	Water	≈2–3 Å	Pristine; water layers.	[29]
Graphene-covered mica; A small piece of graphite flake from HOPG (ZYB grade) was taken with tweezers and placed/pressed between two mica plates. The top mica plate was removed; the graphite flake was mechanically exfoliated using tweezers to obtain a graphene-covered mica surface.	Water (MilliQ, ≈18.2 MΩ·cm)	≈3–6 Å	Mixed; two types of structures:<br>1. No hydration on top of ordered structures<br>2. Hydration layers on clean graphene	
Graphite (HOPG purchased from SPI supplies; freshly cleaved immediately before each experiment)	Phosphate buffered saline (PBS) titrated to pH≈7.3; non-degassed, degassed, or degassed and	Degassed: ≈3.2 Å; Non-degassed: ≈10 Å; Nitrogen-supersaturated: > ≈4 nm	Non-pristine; hydrophobic surface–water interface is depleted of water and dominated by gas layers. These condensed gas layers can be removed and replaced by water layers by degassing the solution; small nanobubbles	[30]

	supersaturated with nitrogen		may be sparsely formed on the surface.	
Perfluoro-decyl-tri-chlorosilane (FDTS) SAM [deposited from vapor phase on a quartz (100) substrate; quartz was purchased from University Wafer]	Phosphate buffered saline (PBS) titrated to pH≈7.3; non-degassed, degassed, or degassed and supersaturated with nitrogen	Degassed: no layers observed; non-degassed or nitrogen-supersaturated: ≈3 nm interfacial long-range force.	Similar conclusion as above. In degassed solutions, the surface is wetted with water; but in other cases, the interface is dominated by condensed gas with a thickness of ≈3 nm.	
HOPG (freshly cleaved). All the other parts of the cell were cleaned in freshly prepared piranha acid and boiled in ultrapure water.	0.1 M H_2SO_4 [prepared from 96% H_2SO_4 (Merck, Suprapur) and ultrapure water (>18 MΩ·cm)]	≈3–4 Å; layer distance decreased from ≈4 Å to ≈3 Å along with more layers observed at very positive potentials.	Pristine; the interfacial liquid region is dominated by water; at more negative potentials, the water layers are more compact and pronounced, possibly due to charge accumulation or adsorption.	31
Graphite (freshly cleaved, HOPG, SPI-2 grade from SPI supplies) under voltage control using a platinum counter and Au/AuO$_x$ reference electrodes.	0.1 M $HClO_4$ in water (ultra-pure from Kanto Kagaku) and 0.1 M H_2SO_4 in water (ultra-pure from Kanto Kagaku);	≈4 Å at −0.9 V, −0.4 V and 0 V; reduced to ≈3 Å at 0.4 V	Pristine; water dominant; voltage and anion dependent interlayer-distance, hydration force and observable number of layers; structure breaking and forming ions modulate the hydration force and longer-range ordering. The origin of the reduction of layer spacing remains unclear.	32
Iodine-modified Au (111) under voltage control using a platinum counter and Au/AuO$_x$ reference electrode. [Au (111) thin films (200 nm thick) on mica were annealed in butane, cooled, and immersed in 1	0.1 M $HClO_4$ in water [prepared using 60% $HClO_4$ (ultrapure, Kanto Chemical) and Milli-Q water]	≈4 Å (at −0.8 V and −0.4 V); only one observable peak at 0 V	At 0 V, the interface becomes enriched with perchlorate ions, disrupting hydrogen bonds in the first water layer. At negative voltages, the lack of potential-dependence indicates that the interfacial water structure remains largely unchanged.	33

mM KI for 15+ minutes]					
Mica (Ted Pella)		Water	≈3–4 Å	Pristine; water dominant.	34
Graphene-covered substrates (mica, quartz and MgO) with different surface charge densities. Before graphene transfer, specific areas of substrate underwent contact electrification by repeatedly scanning the sample with sharp tip in contact mode.	Mica	Either pure water or 50 mM NaCl in water (NaCl purchased from Alladin)	Neutral: d_{01}: 2.6 Å d_{12}: 3.4 Å; positively charged: d_{01}: 1.4 Å d_{12}: 3.3 Å; negatively charged: d_{01}: 1.9 Å d_{12}: 4.9 Å	Pristine; attributed to water layers; both substrate and surface charge play a critical role in the hydration layer structure. Overall, the structure can be strengthened, distorted or collapsed through dynamic interplay between water dipoles and the adsorbed ion species.	
	Quartz		Neutral: d_{01}: 2.3 Å d_{12}: 3.8 Å positively charged: d_{01}: 2.0 Å d_{12}: 4.7 Å negatively charged: d_{01}: 3.3 Å d_{12}: 3.2 Å		
	MgO		Neutral: d_{01}: 3.3 Å d_{12}: 3.0 Å positively charged: d_{01}: 2 Å negatively charged: d_{01}: 1.8 Å		
Graphite (HOPG grade B from Bruker) with voltage control		50 mM NaCl (NaCl purchased from Alladin)	d_{01} is voltage dependent; d_{12}: ≈5 Å; d_{23}: ≈5 Å	Non-pristine; The larger spacing of ~ 5 Å was attributed to the presence of hydrocarbons. The distance between first layer and substrate (d_{01}) decreased from 4.5 to 1.9 Å with change in potential from −0.3 and 0.3 V; from 0.3 V to	

			0.9 V the spacing increased from 1.9 to 5 Å.	
Mica	Water (deionized water, degassed)	≈4 Å	Pristine; water dominant.	[35]
Graphite	Water (deionized water, degassed)	≈3 Å	Pristine; water dominant.	
Graphite	1 M NaCl in water (deionized water, degassed)	Only one observable layer	The AFM tip may not have reached the substrate surface; the ions may strengthen the first observed water layer.	
SAM of Perfluoro-decyl-trichloro-silane (FDTS) on amorphous silica substrate	Water (deionized water, degassed)	No layers observed	Water depletion near the surface.	
Silica with either OTS or 3-amino-propyltriethoxy-silane (APTES) SAMs	1 mM KCl in water (water used is ultrapure, 18.2 MΩ cm, pH ≈ 5.6)	≈4.4–4.8 Å and ≈2.7–3.3 Å for OTS and APTES, respectively	Hydrophobic substrate–water interfaces primarily consist of adsorbed straight-chain alkane layers, whereas hydrophilic substrate–water interfaces are predominantly composed of water layers.	[36]
HOPG (ZYB grade, Bruker) freshly cleaved with tape before the experiment	Ultrapure water (18.2 MΩ cm), pH ≈ 5.6	≈3 Å; increased to 5 Å after ≈50 minutes and remained stable	Hydration layers transitioned to hydrocarbon-dominant layers at the interface upon air exposure.	[7]
Muscovite mica (Grade V-1, SPI supplies, USA)	200 mM KCl in water (KCl: >99%, Sigma-Aldrich; ultrapure water: 18.2 MΩ cm)	≈3–3.5 Å	Pristine, water dominant hydration layers.	[37]
HOPG (ZYB, Bruker) and mica (Grade V-1, SPI)	Ultrapure water (18.2 MΩ cm), pH ≈ 5.6	≈3 Å	Pristine, water dominant	[38]

Various 2D materials(monolayer graphene, few-layer MoS_2, WSe_2, WS_2, and $MoSe_2$) deposited on silicon/silica surface.<br><br>Bulk substrate (thick): HOPG, mica, WSe_2, MoS_2; cleaned by tape exfoliation after immersion in water	Water (ultrapure, pH ≈ 5.6); 200 mM KCl in water (on mica substrate)	For all 2D materials:<br>d_{01}: ≈3–3.5 Å<br>d_{12}: ≈4.5–5 Å<br>d_{23}: ≈5–6 Å<br><br>Mica:<br>d_{01}: ≈2 Å<br>d_{12}: ≈3.4 Å<br><br>Graphite:<br>d_{01}: ≈4.4 Å<br>d_{12}: ≈5.5 Å	Non-pristine; the larger interlayer distance at hydrophobic substrate/water interface could either be attributed to hydrocarbon contaminants or condensed gas molecules.	[39]
Muscovite mica (SPI supplies)	0.2 M NaCl in water; 0.01–0.1 M and 4 M KCl in water; 6 M RbCl in water; ultrapure water	≈3 Å for low molarity solution (0.01 M–0.2 M); ≈5 Å for high molarity (>3 M).	At low concentration, the interface consists of pristine hydration layers with adsorbed cations on the substrate surface. At high concentration, alternating cation and anion layers occur.	[40]
Mica	200 mM KCl in water	≈3 Å	Pristine; hydration layers	[41]
Graphite and mica	Water	Mica: ≈2.2 Å<br>Graphite: ≈3.2 Å	Pristine; the difference in layer spacing was attributed to hydrophobic/hydrophilic effects.	[42]
Lipid bilayer (dipalmitoylphosphatidylcholine, DPPC) supported on mica	Water	≈3.4–3.8 Å	Pristine; hydration layers	[43]
SAM of sulfonic acid (SA)- and trimethylamine (TMA)-terminated thiols supported on gold	Phosphate buffer saline (PBS) solution	2–3 Å	Pristine; hydration layers	[44]
Dolomite [$CaMg(CO_3)_2$]	Pure water (Millipore)	≈3 Å	Pristine; hydration layers	[45]
Calcite ($CaCO_3$)	Pure water	≈2–3 Å	Pristine; hydration layers	[46]

Albite	100 mM KCl in water	≈2–3 Å on clean surface; ≈5 Å on adsorbates	Mixed; pristine hydration layers showing ~2–3 Å layer spacing whereas contaminants at the surface induce ~5 Å spacing.	[47]

Supplementary Table 4 | Summary of studies employing spectroscopy methods to probe solid–water interfaces

Solid substrate	Liquid	Method	Key observations	Ref.
OTS monolayer on fused quartz	Water with pH control (using NaOH or HCl)	Phase sensitive sum frequency vibrational spectroscopy	The neat water/OTS interface is not neutral but charged with adsorbed OH^- ions.<br>The interface consists of DAA and DDA type water molecules at the innermost layer and DDAA water in the subphase layer (D: donor; A: acceptor).	48
Pd (111) monolayer on Au (with voltage control)	0.1 M $NaClO_4$ in water	SHINERS	≈3150 cm^{-1}: 4-HB water; ≈3350 cm^{-1}: 2-HB water; ≈3550 cm^{-1}: Na-H_2O;<br>Potential dependence: As the potential decreases from 0.29 V to −1.11 V (vs. RHE), the population of 4-HB water declines, 2-HB water remains relatively stable, and Na-H_2O shows an increase.	49
Au (111) (with voltage control)	0.1 M Na_2SO_4 in water	SHINERS	≈3300 cm^{-1}: tetrahedral HB; ≈3450 cm^{-1}: trihedral HB; ≈3600 cm^{-1}: dangling OH<br>Potential dependence: interfacial water molecules reorient from a parallel configuration to one-H-down, and then to two-H-down as the potential is reduced from −0.49 V to −2.13 V (vs. PZC).	50
Single-layer-graphene to the surface of Au(111) (with voltage control)	0.1 M $NaClO_4$ in water	SHINERS	≈3200 cm^{-1}: 4-HB water; ≈3400 cm^{-1}: 2-HB water; ≈3550 cm^{-1}: Na-H_2O<br>Potential dependence: As the potential decreases from −0.6 V to −1.5 V (vs. SCE), the amount of 4-HB water remains to be constant, while 2-HB water decreases and Na-H2O increases.	51
Au (~20 nm Au film evaporated on silicon-nitride membrane) (with voltage control)	10 µM NaCl in water	X-ray absorption spectroscopy (XAS)	Four types of water species: Non-donor, single-donor parallel and perpendicular to the surface, and double-donor.<br>Potential dependence: Under more negative bias, water molecules reorient with their hydrogen atoms facing the surface, leading to a reduction in double-donor species and an increase in single-donor species.	52
Au covered with hexadecanethiol monolayer (with voltage control)	10 µM NaCl	XAS		

Au (111) (with voltage control): 20 nm thick gold film coated on a silicon substrate, consisting of small crystallites with (111) surfaces.	0.5 M $HClO_4$ in water	Surface-enhanced infrared absorption spectroscopy (SEIRAS)	Hydrocarbons: ≈2920 cm^{-1}; ≈2850 cm^{-1}.<br>Water (3000–3650 cm^{-1}): ≈3250 cm^{-1} due to Fermi resonance between HOH bending and OH stretching; ≈3400 cm^{-1} from strongly H-bonded water; ≈3500 cm^{-1} from weaker H-bonding; ≈3600 cm^{-1} from non-H-bonded OH.<br>Potential dependence: Interfacial water molecules are weakly H-bonded below the potential of zero charge (pzc), yet strongly H-bonded above pzc forming ice-like structures; at high positive potentials, the ice-like structure is broken by perchlorate ions.	[53]

Supplementary Table 5 | 3D-AFM probe parameters (Urbana)

**Experiment date**	**Liquid**	**Corresponding figures**	**Cantilever type**	**Spring constant (nN nm^{-1})**	**Amp. InvOLS (nm V^{-1})**	***Q* factor**	**Res. freq. (kHz)**
Apr 01, 2021 (set U4)	0.1 M K_2SO_4	Supplementary Figs. 4b, 8	FS-1500AuD	16.92	11.85	6	725
Mar 30, 2022 (set U2)	0.01 M K_2SO_4	Supplementary Figs. 4b, 6, 12a	PPP-NCHAuD	40.66	25.87	9.4	143
Jul 19, 2022 (set U1)	0.01 M K_2SO_4	Fig. 3a, Supplementary Figs. 4b, 5	PPP-NCHAuD	39.12	27.07	9.3	143
Dec 13, 2022 (set U3)	0.01 M K_2SO_4	Supplementary Figs. 4b, 7, 12b	PPP-NCHAuD	38.14	25.43	9.1	137
May 03, 2023 (set U5)	0.01 M K_2SO_4	Supplementary Fig. 9	PPP-NCHAuD	36.78	25.54	9.2	140.5
Apr 27, 2021 (set U6)	0.1 M K_2SO_4	Supplementary Figs. 4b, 10, 16a	FS-1500AuD	15.34	12.52	3.9	629

Supplementary Table 6 | 3D-AFM probe parameters (Madrid)

Experiment date	Liquid	Corresponding figures	Cantilever type	Spring constant (nN nm^{-1})	Amp. InvOLS (nm V^{-1})	*Q* factor	Res. freq. (kHz)
Mar 02, 2022 (set M1)	Pure water	Fig. 2b (pure water panel), Supplementary Figs. 3a, 4a	Arrow-UHFAuD	9.0	17.74	7.4	674.1
Jun 05, 2022 (set M2)	Pure water	Supplementary Fig. 3b	Arrow-UHFAuD	7.8	21.53	8.0	800.8
Jun 27, 2022 (set M3)	Pure water	Supplementary Fig. 3c, 4a	Arrow-UHFAuD	9.3	29.42	8.4	803.1
Oct 10, 2023 (set M7)	0.01 M K_2SO_4	Supplementary Fig. 11c	Arrow-UHFAuD	8.3	40.54	9.4	703.1
Oct 12, 2023 (set M6)	0.01 M K_2SO_4	Supplementary Figs. 4a, 11b	Arrow-UHFAuD	8.9	31.87	4.0	799.5
Sep 06, 2023 (set M9)	0.2 M K_2SO_4	Supplementary Figs. 4a, 13b	Arrow-UHFAuD	7.1	24.37	5.4	772.7
Jan 20, 2024 (set M8)	0.2 M K_2SO_4	Fig. 2b (0.2 M K_2SO_4 panel), Supplementary Figs. 13a, 14b	Arrow-UHFAuD	7.8	58.02	5.0	646.3
Feb 22, 2024 (set M10)	0.1 M KCl	Supplementary Figs. 4a, 13c	Arrow-UHFAuD	8.3	32.70	6.4	858.9
Feb 27, 2024 (set M5)	0.1 M KCl	Supplementary Fig. 11a	Arrow-UHFAuD	9.3	19.57	3.6	984.3
Jul 10, 2024 (set M4)	0.1 M KCl	Figs. 2b (0.1 M KCl panel), 4, Supplementary Figs. 3d, 14a	Arrow-UHFAuD	7.6	22.49	5.5	696.1

Supplementary Table 7 | 3D-AFM imaging parameters (Urbana)

Experiment date	Sample	Corresponding figures	Sample volume (µL)	z rate (Hz)	Sampling rate (kHz)	Free amplitude (pm)	Amplitude setpoint (pm)
Apr 01, 2021 (set U4)	0.1 M K_2SO_4	Supplementary Figs. 4b, 8	100–150	10	500	90–100	60–80
Mar 30, 2022 (set U2)	0.01 M K_2SO_4	Supplementary Figs. 4b, 6, 12a	100–150	10	500	150–160	40–50
Jul 19, 2022 (set U1)	0.01 M K_2SO_4	Fig. 3a, Supplementary Figs. 4b, 5	100–150	10	500	120–140	40–50
Dec 13, 2022 (set U3)	0.01 M K_2SO_4	Supplementary Figs. 4b, 7, 12b	100–150	10	500	100–130	20–40
May 03, 2023 (set U5)	0.01 M K_2SO_4	Supplementary Fig. 9	100–150	10	500	120–150	40–60
Apr 27, 2021 (set U6)	0.1 M K_2SO_4	Supplementary Figs. 4b, 10, 16a	100–150	10	500	70–80	40–60

Supplementary Table 8 | 3D-AFM imaging parameters (Madrid)

Experiment date	Sample	Corresponding figures	Sample volume (µL)	z rate (Hz)	Samp ling rate (kHz)	Free amplitude (pm)	Amplitude setpoint (pm)
Mar 02, 2022 (set M1)	Pure water	Fig. 2b (pure water panel), Supplementary Figs. 3a, 4a	20	100	50	100–150	70–80
Jun 05, 2022 (set M2)	Pure water	Supplementary Fig. 3b	20	100	50	100–150	70–80
Jun 27, 2022 (set M3)	Pure water	Supplementary Figs. 3c, 4a	20	100	50	100–150	70–80
Oct 10, 2023 (set M7)	0.01 M K_2SO_4	Supplementary Fig. 11c	20	100	50	150–200	100
Oct 12, 2023 (set M6)	0.01 M K_2SO_4	Supplementary Figs. 4a, 11b	20	100	50	150–200	100
Sep 06, 2023 (set M9)	0.2 M K_2SO_4	Supplementary Figs. 4a, 13b	20	100	50	200	140
Jan 20, 2024 (set M8)	0.2 M K_2SO_4	Fig. 2b (0.2 M K_2SO_4 panel), Supplementary Figs. 13a, 14b	100	100	50	150–160	90–100
Feb 22, 2024 (set M10)	0.1 M KCl	Supplementary Figs. 4a, 13c	100	100	50	150–200	100
Feb 27, 2024 (set M5)	0.1 M KCl	Supplementary Fig. 11a	100	100	50	150–200	100
Jul 10, 2024 (set M4)	0.1 M KCl	Figs. 2b (0.1 M KCl panel), 4, Supplementary Figs. 3d, 14a	100	100	50	130–150	80–90

Supplementary References

1. Normal contact of elastic solids – Hertz theory. in *Contact Mechanics* (ed. Johnson, K. L.) 84–106 (Cambridge University Press, Cambridge, 1985).
2. Hopcroft, M. A., Nix, W. D. & Kenny, T. W. What is the Young's modulus of silicon? *J. Microelectromechanical Syst.* **19**, 229–238 (2010).
3. Meng, X. *et al.* Broad modulus range nanomechanical mapping by magnetic-drive soft probes. *Nat. Commun.* **8**, 1944 (2017).
4. Bosak, A., Krisch, M., Mohr, M., Maultzsch, J. & Thomsen, C. Elasticity of single-crystalline graphite: inelastic X-ray scattering study. *Phys. Rev. B* **75**, 153408 (2007).
5. Bard, A. J. & Faulkner, L. R. *Electrochemical Methods: Fundamentals and Applications*. (Wiley Global Education, 2012).
6. Gubskaya, A. V. & Kusalik, P. G. The total molecular dipole moment for liquid water. *J. Chem. Phys.* **117**, 5290–5302 (2002).
7. Arvelo, D. M., Uhlig, M. R., Comer, J. & García, R. Interfacial layering of hydrocarbons on pristine graphite surfaces immersed in water. *Nanoscale* **14**, 14178–14184 (2022).
8. Xia, H., Xiahou, Y., Zhang, P., Ding, W. & Wang, D. Revitalizing the Frens method to synthesize uniform, quasi-spherical gold nanoparticles with deliberately regulated sizes from 2 to 330 nm. *Langmuir* **32**, 5870–5880 (2016).
9. Haiss, W., Thanh, N. T. K., Aveyard, J. & Fernig, D. G. Determination of size and concentration of gold nanoparticles from UV−vis spectra. *Anal. Chem.* **79**, 4215–4221 (2007).
10. Li, J. F. *et al.* Shell-isolated nanoparticle-enhanced Raman spectroscopy. *Nature* **464**, 392–395 (2010).
11. Galloway, T. A., Cabo-Fernandez, L., Aldous, I. M., Braga, F. & Hardwick, L. J. Shell isolated nanoparticles for enhanced Raman spectroscopy studies in lithium–oxygen cells. *Faraday Discuss.* **205**, 469–490 (2017).
12. Galloway, T. A. & Hardwick, L. J. Utilizing in situ electrochemical SHINERS for oxygen reduction reaction studies in aprotic electrolytes. *J. Phys. Chem. Lett.* **7**, 2119–2124 (2016).
13. Zheng, Y. *et al.* Toward design of synergistically active carbon-based catalysts for electrocatalytic hydrogen evolution. *ACS Nano* **8**, 5290–5296 (2014).
14. Cheng, L., Fenter, P., Nagy, K. L., Schlegel, M. L. & Sturchio, N. C. Molecular-scale density oscillations in water adjacent to a mica surface. *Phys. Rev. Lett.* **87**, 156103 (2001).
15. Zhou, H. *et al.* Understanding controls on interfacial wetting at epitaxial graphene: experiment and theory. *Phys. Rev. B* **85**, 035406 (2012).
16. Fenter, P., Kerisit, S., Raiteri, P. & Gale, J. D. Is the calcite–water interface understood? Direct comparisons of molecular dynamics simulations with specular X-ray reflectivity data. *J. Phys. Chem. C* **117**, 5028–5042 (2013).
17. Geissbühler, P. *et al.* Three-dimensional structure of the calcite–water interface by surface X-ray scattering. *Surf. Sci.* **573**, 191–203 (2004).

18. Fenter, P. & Sturchio, N. C. Calcite (104)–water interface structure, revisited. *Geochim. Cosmochim. Acta* **97**, 58–69 (2012).

19. Zhang, Z. *et al.* Structure of rutile TiO_2 (110) in water and 1 molal Rb^+ at pH 12: inter-relationship among surface charge, interfacial hydration structure, and substrate structural displacements. *Surf. Sci.* **601**, 1129–1143 (2007).

20. Poynor, A. *et al.* How water meets a hydrophobic surface. *Phys. Rev. Lett.* **97**, 266101 (2006).

21. Jensen, T. R. *et al.* Water in contact with extended hydrophobic surfaces: direct evidence of weak dewetting. *Phys. Rev. Lett.* **90**, 086101 (2003).

22. Toney, M. F. *et al.* Voltage-dependent ordering of water molecules at an electrode–electrolyte interface. *Nature* **368**, 444–446 (1994).

23. Toney, M. F. *et al.* Distribution of water molecules at Ag(111)/electrolyte interface as studied with surface X-ray scattering. *Surf. Sci.* **335**, 326–332 (1995).

24. Schwendel, D. *et al.* Interaction of water with self-assembled monolayers: neutron reflectivity measurements of the water density in the interface region. *Langmuir* **19**, 2284–2293 (2003).

25. Doshi, D. A., Watkins, E. B., Israelachvili, J. N. & Majewski, J. Reduced water density at hydrophobic surfaces: effect of dissolved gases. *Proc. Natl. Acad. Sci.* **102**, 9458–9462 (2005).

26. Maccarini, M. *et al.* Density depletion at solid−liquid interfaces: a neutron reflectivity study. *Langmuir* **23**, 598–608 (2007).

27. Steitz, R. *et al.* Nanobubbles and their precursor layer at the interface of water against a hydrophobic substrate. *Langmuir* **19**, 2409–2418 (2003).

28. Teshima, H., Li, Q.-Y., Takata, Y. & Takahashi, K. Gas molecules sandwiched in hydration layers at graphite/water interfaces. *Phys. Chem. Chem. Phys.* **22**, 13629–13636 (2020).

29. Yang, C.-W., Miyazawa, K., Fukuma, T., Miyata, K. & Hwang, I.-S. Direct comparison between subnanometer hydration structures on hydrophilic and hydrophobic surfaces via three-dimensional scanning force microscopy. *Phys. Chem. Chem. Phys.* **20**, 23522–23527 (2018).

30. Schlesinger, I. & Sivan, U. Three-dimensional characterization of layers of condensed gas molecules forming universally on hydrophobic surfaces. *J. Am. Chem. Soc.* **140**, 10473–10481 (2018).

31. Auer, A., Eder, B. & Giessibl, F. J. Electrochemical AFM/STM with a qPlus sensor: a versatile tool to study solid-liquid interfaces. *J. Chem. Phys.* **159**, 174201 (2023).

32. Utsunomiya, T., Yokota, Y., Enoki, T. & Fukui, K. Potential-dependent hydration structures at aqueous solution/graphite interfaces by electrochemical frequency modulation atomic force microscopy. *Chem. Commun.* **50**, 15537–15540 (2014).

33. Utsunomiya, T., Tatsumi, S., Yokota, Y. & Fukui, K. Potential-dependent structures investigated at the perchloric acid solution/iodine modified Au(111) interface by electrochemical frequency-modulation atomic force microscopy. *Phys. Chem. Chem. Phys.* **17**, 12616–12622 (2015).

34. Tang, Z., Lin, S. & Wang, Z. L. Unveiling contact-electrification effect on interfacial water oscillation. *Adv. Mater.* **36**, 2407507 (2024).

35. Xu, Z., Li, H. & Ma, M. Molecular mechanisms of solvation force for aqueous systems. *Nano Lett.* **24**, 16239–16244 (2024).

36. Arvelo, D. M., Comer, J., Schmit, J. & Garcia, R. Interfacial water is separated from a hydrophobic silica surface by a gap of 1.2 nm. *ACS Nano* **18**, 18683–18692 (2024).

37. Hernández-Muñoz, J. *et al.* Subnanometer interfacial forces in three-dimensional atomic force microscopy: water and octane near a mica surface. *J. Phys. Chem. C* **124**, 26296–26303 (2020).

38. Uhlig, M. R. & Garcia, R. In situ atomic-scale imaging of interfacial water under 3D nanoscale confinement. *Nano Lett.* **21**, 5593–5598 (2021).

39. Uhlig, M. R., Martin-Jimenez, D. & Garcia, R. Atomic-scale mapping of hydrophobic layers on graphene and few-layer MoS_2 and WSe_2 in water. *Nat. Commun.* **10**, 2606 (2019).

40. Martin-Jimenez, D., Chacon, E., Tarazona, P. & Garcia, R. Atomically resolved three-dimensional structures of electrolyte aqueous solutions near a solid surface. *Nat. Commun.* **7**, 12164 (2016).

41. Martin-Jimenez, D. & Garcia, R. Identification of single adsorbed cations on mica–liquid interfaces by 3D force microscopy. *J. Phys. Chem. Lett.* **8**, 5707–5711 (2017).

42. Suzuki, K., Oyabu, N., Kobayashi, K., Matsushige, K. & Yamada, H. Atomic-resolution imaging of graphite–water interface by frequency modulation atomic force microscopy. *Appl. Phys. Express* **4**, 125102 (2011).

43. Asakawa, H., Yoshioka, S., Nishimura, K. & Fukuma, T. Spatial distribution of lipid headgroups and water molecules at membrane/water interfaces visualized by three-dimensional scanning force microscopy. *ACS Nano* **6**, 9013–9020 (2012).

44. Araki, Y., Sekine, T., Chang, R., Hayashi, T. & Onishi, H. Molecular-scale structures of the surface and hydration shell of bioinert mixed-charged self-assembled monolayers investigated by frequency modulation atomic force microscopy. *RSC Adv.* **8**, 24660–24664 (2018).

45. Söngen, H. *et al.* Chemical identification at the solid–liquid interface. *Langmuir* **33**, 125–129 (2017).

46. Söngen, H. *et al.* Resolving point defects in the hydration structure of calcite (10.4) with three-dimensional atomic force microscopy. *Phys. Rev. Lett.* **120**, 116101 (2018).

47. Umeda, K., Kobayashi, K., Minato, T. & Yamada, H. Atomic-scale 3D local hydration structures influenced by water-restricting dimensions. *Langmuir* **34**, 9114–9121 (2018).

48. Tian, C. S. & Shen, Y. R. Structure and charging of hydrophobic material/water interfaces studied by phase-sensitive sum-frequency vibrational spectroscopy. *Proc. Natl. Acad. Sci.* **106**, 15148–15153 (2009).

49. Wang, Y.-H. *et al.* In situ Raman spectroscopy reveals the structure and dissociation of interfacial water. *Nature* **600**, 81–85 (2021).

50. Li, C.-Y. *et al.* In situ probing electrified interfacial water structures at atomically flat surfaces. *Nat. Mater.* **18**, 697–701 (2019).

51. Wang, Y.-H. *et al.* Characterizing surface-confined interfacial water at graphene surface by in situ Raman spectroscopy. *Joule* **7**, 1652–1662 (2023).

52. Velasco-Velez, J.-J. *et al.* The structure of interfacial water on gold electrodes studied by X-ray absorption spectroscopy. *Science* **346**, 831–834 (2014).

53. Ataka, K., Yotsuyanagi, T. & Osawa, M. Potential-dependent reorientation of water molecules at an electrode/electrolyte interface studied by surface-enhanced infrared absorption spectroscopy. *J. Phys. Chem.* **100**, 10664–10672 (1996).
