# Supplementary material for: Probing the molecular structure at graphite–water interfaces by correlating 3D-AFM and SHINERS
Source: Nat Commun. 2026 Jan 31;17:2230. doi: 10.1038/s41467-026-68667-y (PMC12963383; doi:10.1038/s41467-026-68667-y)
Supplement: Supplementary file 2 — Description of Additional Supplementary Files [file 41467_2026_68667_MOESM2_ESM.pdf]

File Name: Supplementary Data 1
Description: Unprocessed spectroscopic data
